# Understanding “Atmosome”, the Personal Atmospheric Exposome: Comprehensive Approach

**DOI:** 10.2196/28920

**Published:** 2021-11-23

**Authors:** Hari Bhimaraju, Nitish Nag, Vaibhav Pandey, Ramesh Jain

**Affiliations:** 1 Donald Bren School of Information and Computer Sciences University of California Irvine, CA United States

**Keywords:** exposome, exposomics, personal health, indoor air quality, health state estimation, health informatics, public health policy, epidemiology, embedded systems, internet of things

## Abstract

**Background:**

Modern environmental health research extensively focuses on outdoor air pollutants and their effects on public health. However, research on monitoring and enhancing individual indoor air quality is lacking. The field of exposomics encompasses the totality of human environmental exposures and its effects on health. A subset of this exposome deals with atmospheric exposure, termed the “atmosome.” The atmosome plays a pivotal role in health and has significant effects on DNA, metabolism, skin integrity, and lung health.

**Objective:**

The aim of this work is to develop a low-cost, comprehensive measurement system for collecting and analyzing atmosomic factors. The research explores the significance of the atmosome in personalized and preventive care for public health.

**Methods:**

An internet of things microcontroller-based system is introduced and demonstrated. The system collects real-time indoor air quality data and posts it to the cloud for immediate access.

**Results:**

The experimental results yield air quality measurements with an accuracy of 90% when compared with precalibrated commercial devices and demonstrate a direct correlation between lifestyle and air quality.

**Conclusions:**

Quantifying the individual atmosome is a monumental step in advancing personalized health, medical research, and epidemiological research. The 2 main goals in this work are to present the atmosome as a measurable concept and to demonstrate how to implement it using low-cost electronics. By enabling atmosome measurements at a communal scale, this work also opens up potential new directions for public health research. Researchers will now have the data to model the impact of indoor air pollutants on the health of individuals, communities, and specific demographics, leading to novel approaches for predicting and preventing diseases.

## Introduction

At any moment in time, health is affected by various internal and external factors, such as the genome, microbiome, and exposome. The exposome consists of everything an individual is exposed to across his or her lifespan [1]. It considers lifestyle, occupation, socioeconomic factors, and the environmental conditions in which people live to develop an in-depth understanding of how an individual’s surroundings impact his/her health. The atmospheric exposome, a subset of the complete exposome and which is presented in this work, focuses on the health effects from the air that people breathe.

The term “atmosome” was coined to describe the atmospheric subset of an individual’s exposome. Common indoor air pollutants include PM_2.5_ (particulate matter with diameter of ≤2.5 μm), PM_10_ (particulate matter with diameter of ≤10 μm), carbon dioxide (CO_2_), nitrogen dioxide (NO_2_), carbon monoxide (CO), volatile organic compounds (VOCs), ozone (O_3_), liquid petroleum gas (LPG), natural gas (NG), formaldehyde (HCHO), and biological contaminants such as bacteria and fungi. Measuring the quality of indoor air can provide insights into the potential adverse effects of poor air quality and preventative measures to keep the exposome cleaner. A cleaner atmosome, in turn, has a positive impact on health and well-being. Thus, there is a need for a portable, real-time, multichannel air measurement system for enabling data-driven analytics and research [2].

A number of studies have been conducted by several federal, state, and local agencies that monitored, collected, and stored outdoor air quality data in the Environmental Protection Agency’s (EPA) Air Quality System database [3,4]. The Environmental Defense Fund in collaboration with Google Earth [5], the World Health Organization Global Urban Ambient Air Pollution Database [6], the World Air Quality Historical Database [7], and many other organizations generated air quality maps. These data are used for various modeling studies, to review policy implementation plans, and to generate reports for the Congress (US) [5]. However, these agencies have overlooked similar quantitative studies of indoor air quality (IAQ).

Previous studies have shown that indoor air is much more polluted than outdoor air and represents a major public health challenge especially in developing countries [8]. Many studies focused on specific or limited indoor air pollutants such as VOCs, CO_2_, PM_2.5_, PM_10_, and halogen flame-retardants [9-12]. Further, though potentially unexpected, there exist a myriad of well-defined sources of indoor air contamination and, correspondingly, numerous contaminants [13]. To improve air quality and minimize pollution-related disease and mortality, the atmosome must be defined, measured, and analyzed to mitigate adverse environmental conditions and improve health outcomes. Thus, the motivation for this work is to use the atmosome to further personalize an estimation of individual health from multimodal data [14,15].

According to the EPA, indoor air pollution is one of the top 5 environmental risks to public health. Annually, 9 out of every 10 people breathe air containing high levels of pollutants at some point [16,17]. Air pollutants can be in the form of pet dander, mold, dust mites, CO, radon, pests, lead, and secondhand smoke [17]. Americans spend approximately 90% of their time indoors, where the concentrations of some airborne pollutants are 2-5 times higher than those outdoors [13]. This poor IAQ can cause various infections, lung cancer, and chronic lung diseases, including asthma [18]. It can also contribute to the development of atherosclerosis, a root cause of many cardiovascular diseases [19]. In 2020, a long-term study of over 63 million US adults indicated a surprising correlation between PM_2.5_ and hospitalizations for severe neurological diseases [20].

According to the State of Global Air 2020 [21], nearly 500,000 newborns died in 2019 in their first month of life due to exposure to all types of air pollutants described previously. Household inhalation of mold spores and infant pulmonary hemorrhage are found to be linked in some studies [22]. Air pollution even impacts children while they are in their mothers’ womb [23,24], with the effect of air pollution on pregnant women and their fetuses comparable to smoking tobacco [21]. People often assume that indoor spaces are safe from outdoor air pollution, but this is inaccurate. Therefore, IAQ is a significant threat to public health [25]. The Program Needs for Indoor Environments Research (PNIER) document details EPA’s research needs for the indoor environment and recommends that the EPA and other governmental and private sector agencies and organizations address this issue [26].

The environmental research field of IAQ is nascent. Nonetheless, several researchers have recently demonstrated portable gas detection systems using various sensor technologies covering a limited set of analytes. For example, MQ sensors were embedded in a system where VOCs were detected [27]. MQ sensors are well-known to exhibit acceptable selectivity, but low sensitivity. Interestingly, the researchers implemented an artificial neural network and dramatically improved the sensitivity to gas concentrations at single-digit parts per million (ppm). However, the supported analyte set is far too small given the contaminants of concern, as reported by the EPA. A related research effort embedded photoionization detectors (PIDs) into a portable system for the detection of isobutylene, ethanol, propanol, and acetone [28]. PID sensors are well-known to exhibit high sensitivity (ie, on the order of parts per billion [ppb]), but lower selectivity than MQ sensors. Further, that work demonstrated only a small set of detected analytes. Other recent efforts include a portable system with embedded gas chromatography PID sensors [29]. This system detects benzene, toluene, and xylene. The researchers also employed an elegant algorithm using various quantification parameters (eg, pumping time, temperature) and calibration curves to optimize selectivity. This system also requires a pumping time up to 90 seconds and an analysis time of 10 minutes. Nonetheless, as expected, very high sensitivity was achieved, but again the analyte set is small and the system cost is substantially higher than an implementation with low-cost devices, such as MQ sensors. Last, none of these research efforts considered management of the data in an actionable manner. The systems reported were to demonstrate selectivity and sensitivity, the 2 most critical metrics for gas sensors.

This recent research focused on utilizing more common sensor technologies for portable applications. However, more exotic sensors have been developed recently in portable gas detection systems that have been demonstrated for indoor and outdoor use. For example, in [30], a mobile microscopy system (coined as the c-Air device) is presented and utilizes microscopy as a sensing technique and includes machine learning algorithms to increase accuracy. Further, it includes a mobile software app for data display. The device requires a sample of 6.5 L of air and an analysis time of 30 seconds. Also, similar to related studies, the c-Air device supports only a limited set of analytes, including total suspended particulates, PM_10_, and PM_2.5_. Nonetheless, very good results are achieved and it is an advancement that the system is linked to a mobile app, though nothing actionable is reported.

In [31], a so-called portable cyber-physical system is presented for gas detection and is embodied in 2 distinct architectures, a stationary and portable device, each using well-established electronics related to this work, including an Arduino microcontroller and Raspberry Pi system-on-chip (SoC), corresponding to each embodiment. The 2 systems also use MQ sensors. However, the only sensors supported by the system are an MQ-4 (a methane sensor) and an MQ-8 (a hydrogen sensor). Further, although the system supports connectivity to the internet, the functionality is to merely upload the data to cloud storage. No manipulation or presentation of the data is reported.

Basic IAQ commercial-off-the-shelf (COTS) products also exist. Xiaomi, for example, offers a countertop product which detects PM_2.5_, total VOC (tVOC), CO_2_, temperature, and humidity [32]. It includes a touch screen and Wi-Fi connectivity. The company also offers handheld products that measure individual analytes, such as PM_2.5_ [33]. Further, Xiaomi offers air purifiers that can be controlled by a mobile phone app and are marketed under the brand Mi. There exist many other related COTS products for IAQ monitoring. Although interesting, these products measure a very small set of analytes and offer minimal, if any, actionable information based on the data collected.

In contrast to both the recent research and commercial work, this paper introduces a patent pending and low-cost embedded system called the Atmosome Measurement System (AMS) [34], which can reliably, accurately, and instantaneously monitor and measure significantly more indoor air pollutants such as PM_2.5_, PM_10_, CO_2_, NO_2_, CO, VOCs, O_3_, LPG, NG, equivalent CO_2_ (eCO_2_), hydrogen as well as environmental parameters including temperature, humidity, pressure, and altitude. This set of analytes was chosen as it represents the primary sources of indoor air pollution as well as the leading causes of adverse impact on human respiratory health, according to the EPA. Further, the presented work is implemented as a scalable and low-cost embedded system utilizing COTS electronics including an Arduino microcontroller, Raspberry Pi SoC, MQ gas sensors, and simple environmental sensors. MQ sensors were selected for low cost, high selectivity, and sufficient sensitivity. Besides, in contrast to previous studies [10,12], AMS is built with the goal of providing a cloud-based infrastructure that stores, analyzes, and presents insights into IAQ and trends that correlate with personal lifestyles. It displays historic and real-time data from multiple sensors in a user-friendly web application, enables users to interpret their data, and recommends environmental changes to improve personal atmosome conditions.

Therefore, the development of a system that can evaluate IAQ by using multiple analytes, process and visualize pollutant data, recommend remediation steps, and be built at an affordable price point is the foundation of this research. The system can be configured with a variety of optional customizations including the frequency at which the users would like to monitor their air quality; their geographic location details including the zip code, city, state, and country; the indoor space details such as home, office, or car; the location within the space such as kitchen, bedroom, garage; and the activity details such as cleaning, cooking, routine. Further, AMS supports representational state transfer (REST) application programming interface (API) to download data for further exploration and analytics. AMS also includes an option for the users to anonymously share their data to further indoor air pollution research. This opens the possibility of developing a public indoor air quality database while maintaining user confidentiality allowing for extended research on indoor air quality, its impacts, and health policy modeling.

Taken together, AMS can be a useful tool for improving public health outcomes as it can provide the necessary data that people need to manage their IAQ in a cost-effective and convenient manner. Moreover, the data can display different atmosomes between 2 neighbors or within neighborhoods of different socioeconomic classes, which can be useful for public health officials or policy researchers that work toward enhancing the health of citizens.

Comparisons of AMS with various COTS products are available in Multimedia Appendix 1 [35-41]. AMS stood out in both number of analytes and cost compared with the nearest COTS product, Aeroqual, that covers multiple analytes [35]. Comparisons are also made with recent studies and those details are available in Multimedia Appendix 2 [42-47]. Aspects such as data sampling duration and pollutant streams in AMS are found to be much more extensive than similar indoor air quality assessments in a college campus [44] and homes in a temperate region of the United States [46]. Comparisons are not made with the cited research because the analyte sets are dramatically smaller than those supported by AMS and there is no actionable interpretation of the data in the studies, because they focus primarily on sensor selectivity and sensitivity, as described previously. Further, few of those efforts included internet connectivity and none included any actionable information based on the data collected.

## Methods

### Study Approach and Design

An experimental approach combining internet of things (IoT) hardware and software development was used to measure air pollutants and air quality metrics. The researchers used AMS to nonintrusively monitor air quality through daily indoor life. AMS provided visuals and recorded trends that could indirectly indicate the relationship between lifestyle and observed pollutant values.

In this work, the researchers used AMS to collect air quality data indoors at home (in Cupertino, California; South Lake Tahoe, California; and Hyderabad, Telangana, India) as well as during local commute in the United States and during an inflight journey from the United States to India. These locations were chosen to monitor and evaluate the performance of AMS in environments associated with distinct indoor air quality profiles and climates. The device, similar to a thermostat placed in a room, is completely noninvasive. To initiate readings, it is powered on with a USB cable (or a wall socket) and AMS software is launched by the user. The studies were conducted, intermittently, between January 2020 and January 2021.

After initial calibration, to ensure the continued accuracy of the measurements made by AMS on an ongoing basis, the sensors were recalibrated every 3 months. Measurements were repeated over similar activities in several different timeframes to better analyze and predict possible relationships between activities and associated pollutants. To control for bias, measurements were also carried out across several recalibrations.

### System Architecture

**Figure 1:**
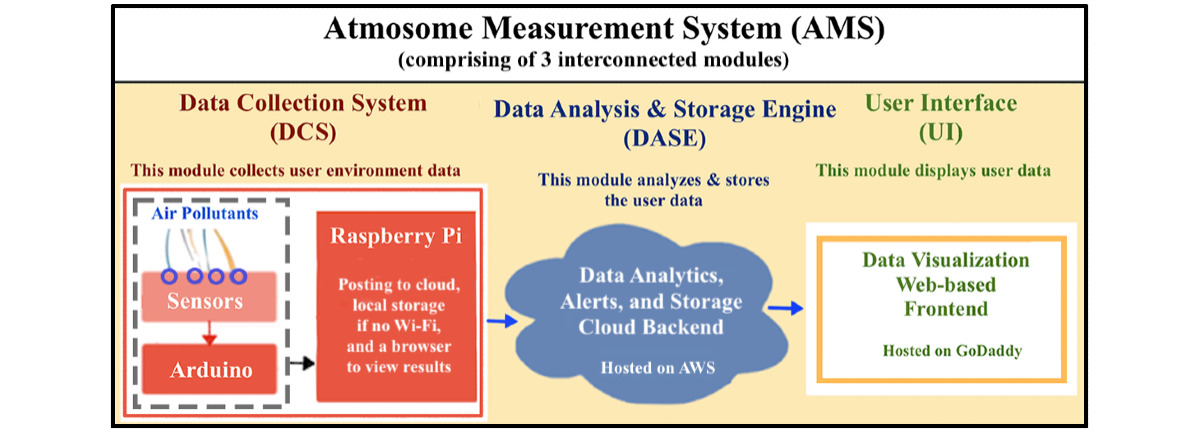
AMS block diagram illustrating the basic functionality for data collection, data analysis and storage, and the user interface.

AMS comprises 3 distinct modules that collect, analyze and store, and display data. As shown in Figure 1, these 3 interconnected modules include the data collection system (DCS), data analysis and storage engine (DASE), and user interface (UI). The DCS is a sensor system that collects the user’s environmental data in user-defined intervals. The data gathered are then analyzed and stored by the system’s DASE, and the air quality metrics obtained are displayed by the system’s web-based UI.

### Materials and Software

Materials used to build the DCS and the software used to create DASE and UI are discussed in the following sections.

#### Data Collection System

##### Overview

The DCS is an IoT air monitoring sensor system for acquiring an individual’s unique geospatial data to track air quality. It includes 17 environmental sensors that measure 22 different air pollutant data streams, an Arduino Mega, a Raspberry Pi, and a power source. Referring back to Figure 1, the Arduino Mega is a microcontroller that captures sensor data and the Raspberry Pi is an SoC which supports Wi-Fi and enables an interface to the cloud.

##### Sensors

The DCS monitors environmental conditions such as temperature, pressure, altitude, humidity, and various analytes including PM_2.5_, PM_10_, CO, O_3_, CO_2_, eCO_2_, tVOCs, LPG, methane, hydrogen, flammable gases, aromatic compounds, hydrogen sulfide, ammonia, nitrogen oxide, NG, and HCHO as shown in Table 1. Metal oxide semiconductor (MOS) gas sensors were used to detect the different air pollutants. MOS-based sensors detect the concentration of various kinds of gases by acting as a chemiresistor, where a change in resistance of the metal oxide occurs due to the adsorption of specific gases. These sensors, and specifically MQ series sensors, are ideally suited for low-cost and low-power applications in indoor environments. Selectivity to certain gases is dependent on the specific sensor model, which is indicated numerically. Some MQ sensors are sensitive to multiple gases; for example, both MQ-5 and MQ-6 measure LPG, but MQ-6 exhibits higher selectivity and sensitivity to LPG and is calibrated for that particular gas. As individual sensors are calibrated for their specific gases, they are less selective to other gases. A total of 17 different kinds of sensors were used to detect and measure the level of pollutants as well as environmental parameters (Table 1).

**Table 1:** List of sensors, including target gas analytes and parameters as well as sensor type, in the data collection system implementation. The system supports 22 data streams from 17 different sensors.

Sensor	Analyte/parameter	Sensor type
CCS811/BME280	Total volatile organic compounds, equivalent carbon dioxide, temperature, humidity, pressure, altitude	Environmental combo sensor
CO_2_	Carbon dioxide	Nondispersive infrared
PM_2.5_	Particulate matter 2.5	Optical, infrared-emitting diode
MQ2	Smoke/particulate matter 10	MOS^a^
MQ4	Methane	MOS gas sensor
MQ6	Liquefied petroleum gas	MOS gas sensor
MQ7	Carbon monoxide	MOS gas sensor
MQ131	Ozone	MOS gas sensor
MQ3	Alcohol (ethyl alcohol)	MOS gas sensor
MQ5	Natural gas	MOS gas sensor
MQ8	Hydrogen	MOS gas sensor
MQ9	Flammable gases	MOS gas sensor
MQ135	Aromatic compounds	MOS gas sensor
MQ136	Hydrogen sulfide	MOS gas sensor
MQ137	Ammonia	MOS gas sensor
NO_x_	Nitrogen oxides	MOS gas sensor
HCHO	Formaldehyde	MOS gas sensor

^a^MOS: metal oxide semiconductor.

##### Realized AMS

**Figure 2:**
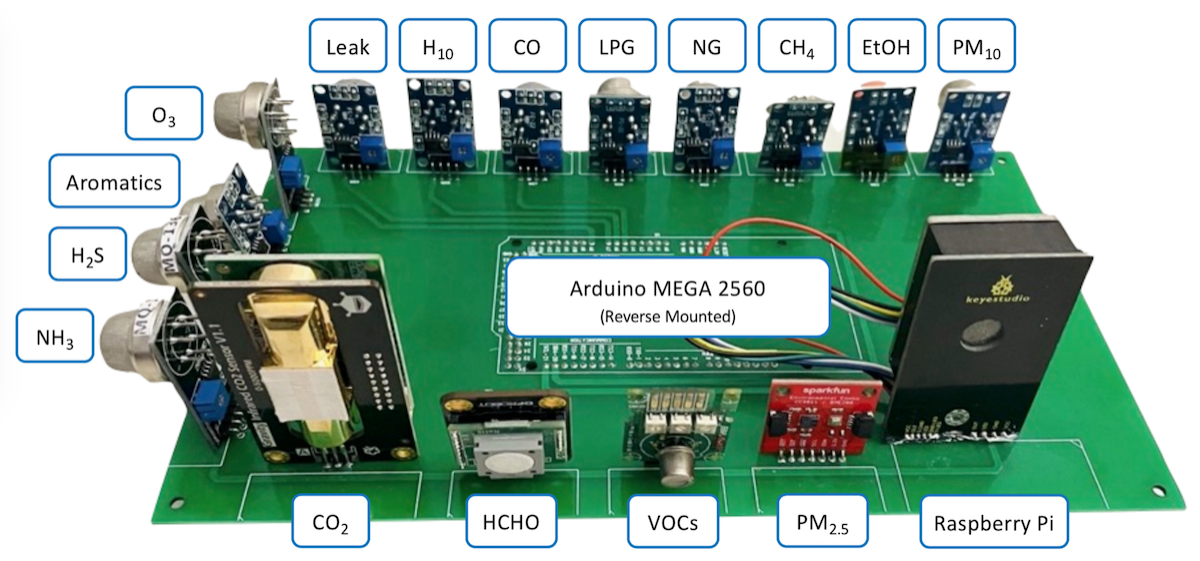
A photograph of the populated PCB with the Arduino (reverse mounted) and 17 sensors listed in Table 1. CO: carbon monoxide; CO_2_: carbon dioxide; H_2_S: hydrogen sulfide; HCHO: formaldehyde; LPG: liquid petroleum gas; NG: natural gas; NH_3_: ammonia; PCB: printed circuit board; PM_2.5_: particulate matter with diameter of 2.5 μm or less; PM_10_: particulate matter with diameter of 10 μm or less; VOC: volatile organic compound.

Figure 2 presents the complete realized AMS including sensors and the Raspberry Pi platform, which are securely mounted to an FR4 printed circuit board (PCB) and an Arduino Mega 2560 microcontroller reverse mounted to the same. The Arduino Mega microcontroller is programmed to capture data from the sensors. All of these system components and associated positions on the PCB are labeled clearly in Figure 2.

##### IoT

The sensor PCB is connected using a USB cable linked to a computer (either a Raspberry Pi or a laptop that runs the DCS software). The computer receives sensor data from Arduino Mega microcontroller and posts it to the cloud (DASE) server or saves it in local memory in the absence of an internet connection. The PCB comes in 2 variations: a portable model that consists of 8 different sensors providing 13 data streams and powered by the USB; and a high-power model that has 17 sensors providing 22 data streams and which must be powered by a wall socket. Future work will aim to make the high-power model portable.

#### Data Storage and Analysis Engine

**Figure 3:**
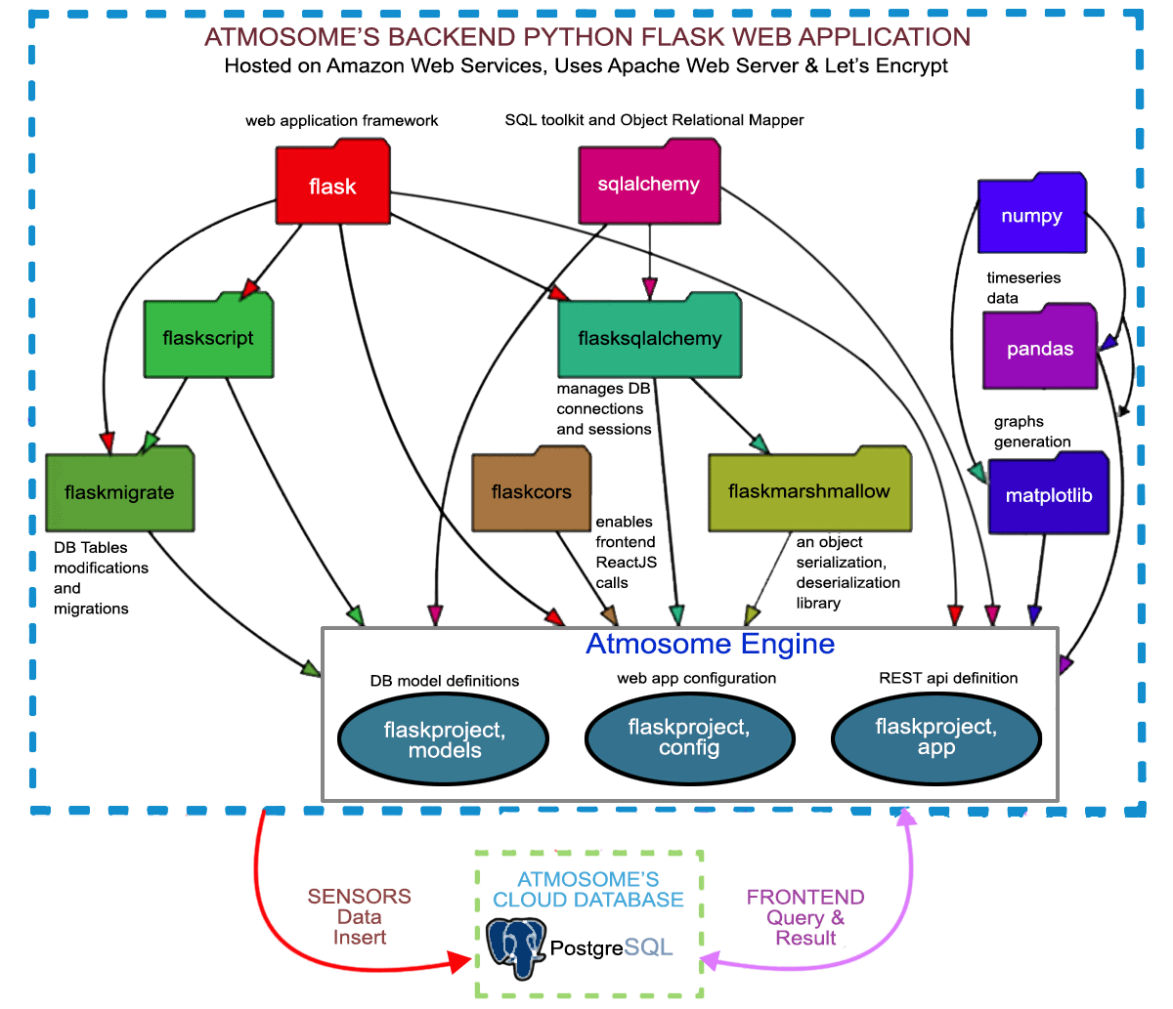
DASE (data analysis and storage engine) programming components and functional flow diagram.

DASE runs in the cloud on Amazon Web Services (AWS) and stores the sensor data in a Postgres Database. AWS was selected for simplicity of implementation, low cost, and well-known user data security as shown in a recent case study [48]. Further, no personally identifiable information is collected for this study. The username and zip code are stored in the cloud and password protected. The software implementation is built using the Python Flask Framework and supports REST API to support receiving data from the sensor board at user location (DCS) and for sending data to the UI or alerts to the user. The source module layout and libraries are shown in Figure 3.

The backend of the system is based on Python and Flask and has 3 distinct interconnected components that make up the Atmosome engine: the web application framework, the database model that receives data from the cloud database, and the numerical analysis libraries that operate on the data received from the user’s device and surface them to the user via the front end UI. To accomplish this workflow, DASE employs various Flask web framework components including *flaskmigrate* (to support modifications to existing DB tables), *sqlalchemy* (an object-relational mapper that enables reading from/writing to the DB python data objects without the need for using the DB’s SQL), *flasksqlalchemy* (to manage DB connections and sessions), *flaskcors* (to enable cross-domain REST API communication between the server and the UI), *flaskmarshmallow* (to handle python-json serialization/deserialization for REST API communication), *numpy* (Python’s multidimensional numerical analysis library), *pandas* (Python’s DataFrame support much like a database table in memory), and *matplotlib* (Python’s data visualization library). *flaskproject.models* defines the DB tables in python, *flaskproject.config* defines the web application configurations, for example, the type of database being used, and *flaskproject.app* is the code that handles all data computations.

#### User Interface

The UI is built as a progressive web application using the React Framework. This makes it available on any device with a web browser. Additionally, because it is a progressive web app, it automatically adjusts to the size of mobile platforms, and thereby presents a user experience similar to a native app. The source code is structured and modularized into pages and components within pages.

**Figure 4:**
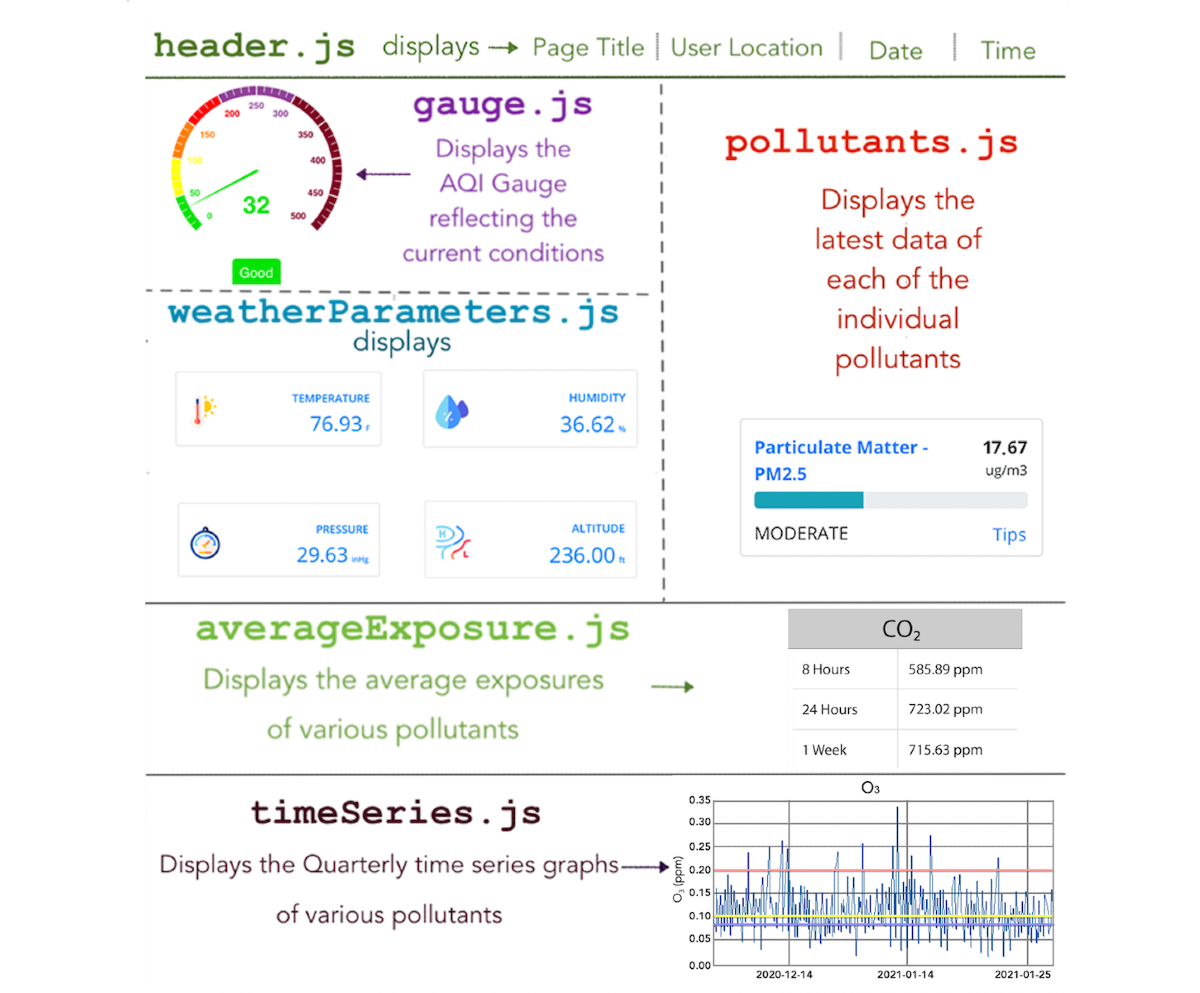
UI dashboard software and display components. The dashboard showcases the level of various pollutants (eg, PM_2.5_, VOC, ozone, CO_2_), weather metrics (eg, temperature, humidity, pressure, and altitude), Air Quality Index, average exposure levels over time, and quarterly temporal graphs reflecting the user’s historic exposure data. CO_2_: carbon dioxide; PM_2.5_: particulate matter with diameter of 2.5 μm or less; UI: user interface; VOC: volatile organic compound.

For example, Figure 4 illustrates the 6 *javascript* (*.js*) source files that render the various sections of the Atmosome dashboard. *header.js* is the header of the page, and displays the page title, user location, date, and time. *gauge.js* displays the AQI gauge which gives an “at-one-glance” state of the current indoor air conditions. *weatherParameters.js* displays the temperature, humidity, pressure, and altitude. Although weather metrics are not pollutants, they are a part of lifestyle conditions and are recorded along with information on pollutants. *pollutants.js* displays numeric and visual information about each of the pollutants. *averageExposure.js* displays the user’s average exposure to various pollutants in different intervals of time. *timeseries.js* displays the quarterly time series graphs of the user’s historic exposure to various pollutants. The UI is designed mainly to render data and does not store data or perform computations. It makes REST API calls to DASE to retrieve the data.

### Procedure

In this section, the methods involved in calibrating the DCS device, collecting data from a setting, and posting data to DASE will be discussed. Moreover, the analyzed data can be viewed on UI screens for the given set up.

#### Sensor Calibration

**Figure 5:**
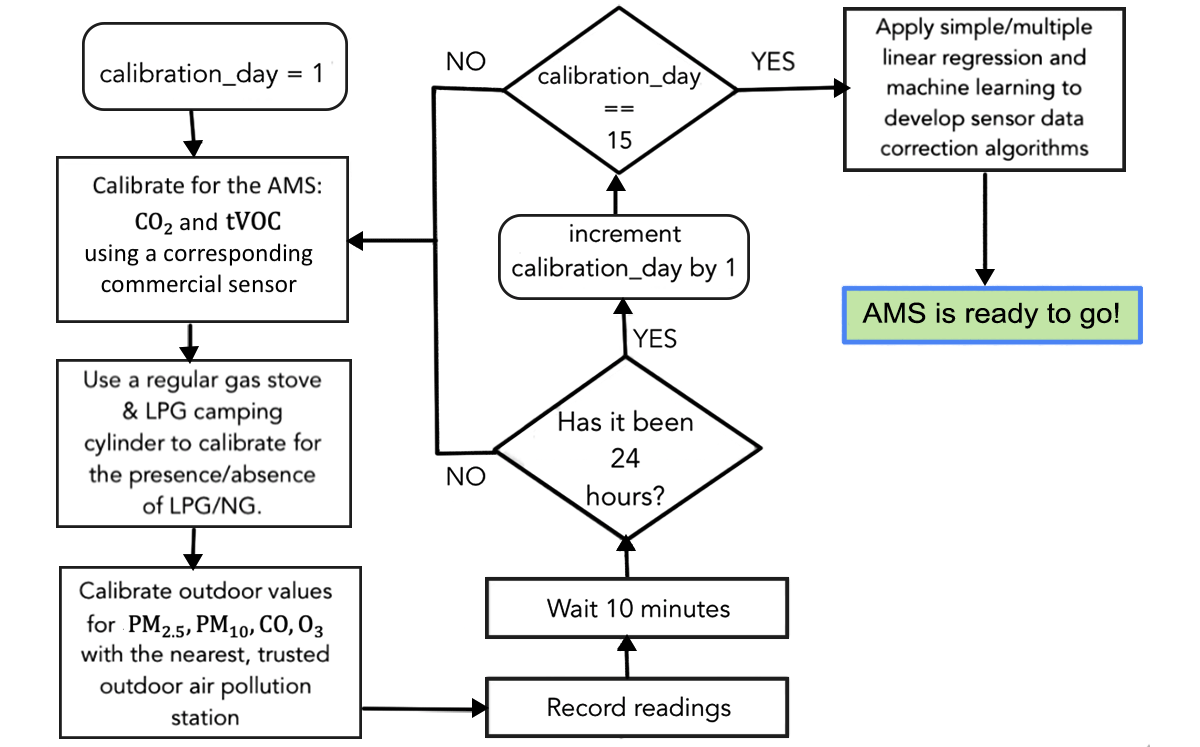
DCS calibration algorithm flow diagram. CO: carbon monoxide; CO_2_: carbon dioxide; DCS: data collection system; LPG: liquefied petroleum gas; NG: natural gas; O_3_: ozone; PM_2.5_: particulate matter with diameter of 2.5 μm or less; PM_10_: particulate matter with diameter of 10 μm or less; tVOC: total volatile organic compound.

The sensors in AMS’s DCS are first “burned-in,” meaning that they are placed in an environment with clean air and operated with active power for 48 hours. Next, AMS’s DCS is run through a meticulous calibration process to ensure accuracy. A flow diagram of the calibration algorithm is illustrated in Figure 5. AMS DCS is calibrated for several variables such as CO_2_, tVOC, LPG, PM_2.5_, PM_10_, and others as indicated. During the initial set up, this process is repeated daily and accumulated over a period of 15 days to train a linear regression model to predict the values. The calibration code is run on the sensor’s board Arduino Mega microcontroller, and the data are accumulated on the Pi system.

To ensure the continued accuracy of the new system, the sensors were recalibrated every 3 months, and new training improves the accuracy of the data correction algorithms. Upon continued comparison of the DCS postcalibration values with precalibrated COTS devices, over 90% accuracy postcalibration was achieved when compared with precalibrated COTS devices. Alternative calibration approaches, such as calibrating outdoors against the values of the nearest outdoor weather station, were explored and similar accuracy was noted.

#### Data Collection

Once the user receives the portable DCS, they need to power it and connect it to a Wi-Fi network, if available. Then they need to launch the application. Once launched, the user can change any of their default settings, or retain the defaults, and initiate collection of air quality data.

Although AMS is intended to measure IAQ, part of the calibration routine was performed outdoors. This decision was made to compare results easily with the nearest, trusted outdoor air pollution station. Further, the environmental differences between the indoor and outdoor settings were not notable enough to introduce a substantial error. Certainly, an entirely indoor calibration routine would yield higher sensor accuracy, but precise indoor calibration techniques are complex and not suitable for consumer use. For example, precision routines to calibrate gas sensors are typically performed in the presence of a high concentration source of each analyte. Such an approach was deemed unrealistic and prohibitively expensive.

The researcher (HB) used AMS to monitor air quality through daily life at home, such as in the kitchen, living room, bedroom, home office, and during commute. The researcher also measured air quality during air travel to India, and at home in India and the United States.

#### Data Transmission

##### Local Storage and Transmission to Cloud

In the presence of Wi-Fi, as the DCS program collects air quality data from the sensors at the frequency specified by the user, it concurrently transmits the data to the cloud and requires no user interaction. In the absence of Wi-Fi, the DCS stores the data in local memory. Once a Wi-Fi connection is available, the user can connect the DCS to the Wi-Fi, and select an icon in the UI that indicates “Upload AMS data to the Cloud.” This loads the data to the cloud, and once complete, deletes the locally stored data, automatically removing the no-longer required data from local storage.

##### Post/Receive Data to/From Cloud

**Figure 6:**
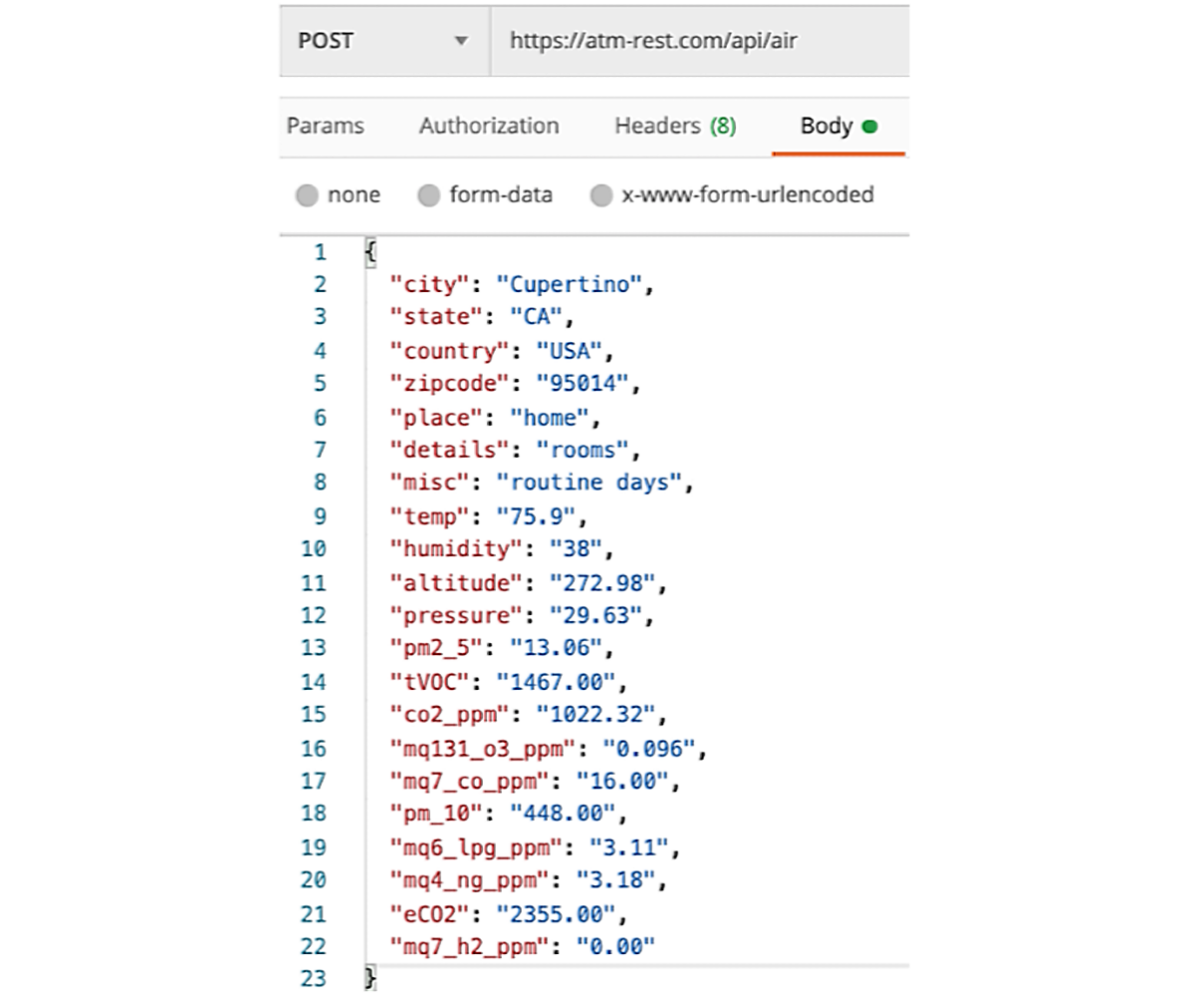
Data transmission: REST API POST data from DCS to DASE containing sensor types and values. API: application programming interface; DASE: DASE: data analysis and storage engine; DCS: data collection system; REST: representational state transfer.

The DCS software wraps the readings from each of its sensors into JSON, a simple format, with a series of key value pairs, used to store and transmit data to DASE, which is running in the cloud. Once a JSON payload with the values of each pollutant, atmospheric data, and sampling location information has been assembled (a portion of which is shown in Figure 6) it is ready to be transmitted via the internet to DASE.

The DCS software uses REST APIs to interact with DASE. REST APIs use HTTP requests to interact with a remote web server. The HTTP GET method is used to receive data from the server, and the HTTP POST method is used to send data to the remote server.

**Figure 7:**
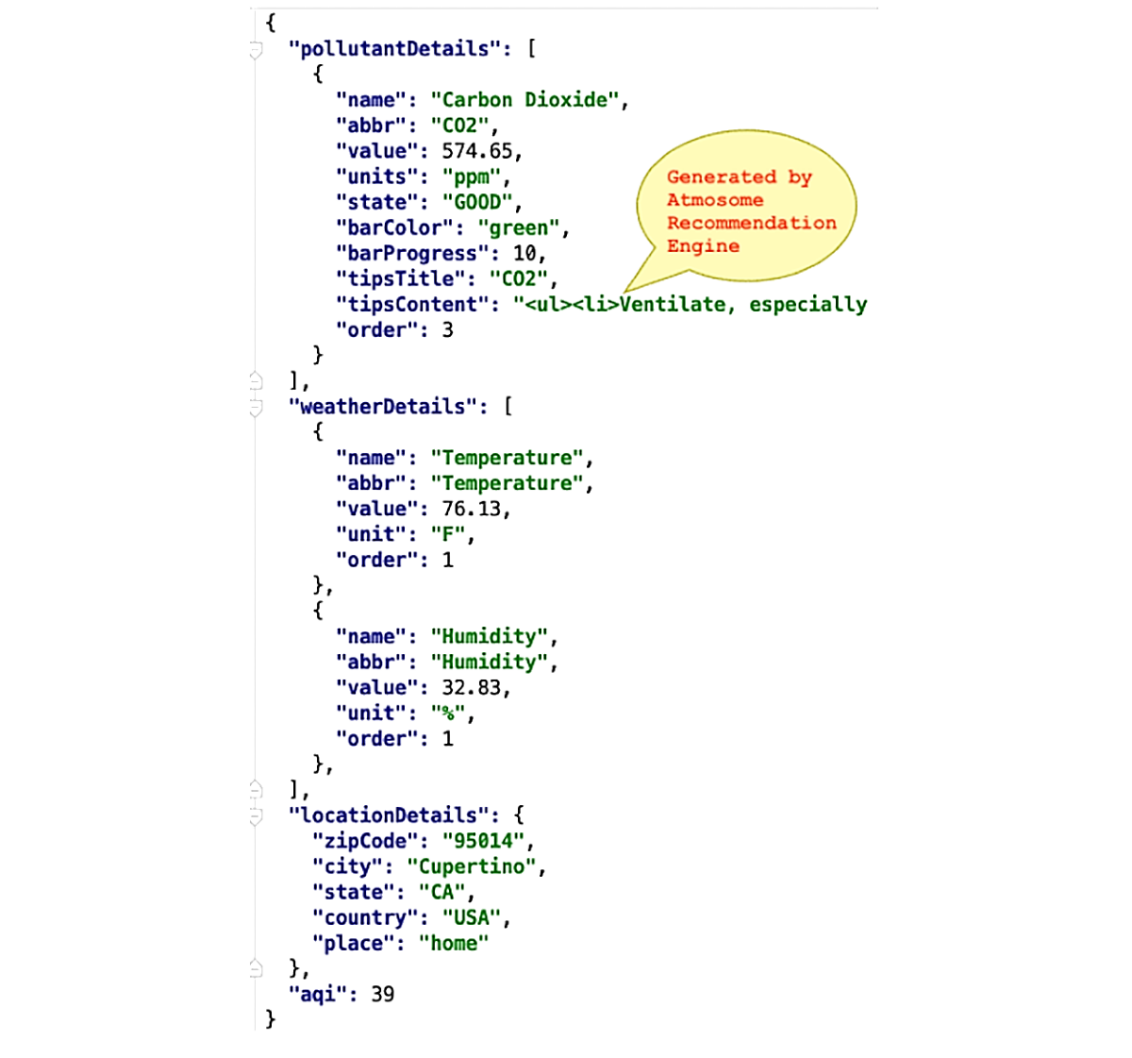
JSON formatted REST API GET data from DASE to UI containing attributes stored for each pollutant and weather metric. API: application programming interface; DASE: data analysis and storage engine; REST: representational state transfer; UI: user interface.

The DCS software sends this JSON payload by making a REST API POST request to DASE, which receives the data, and subsequently analyzes and stores them. Upon receiving an HTTP GET request from the UI to display data, DASE formats the information required by the UI into the JSON payload and sends it, as illustrated in Figure 7. The UI then displays these data.

#### Data Storage

DASE stores the received sensors data in its Postgres database. Postgres is a free database and is seamlessly integrated into DASE’s Python Flask framework to store and retrieve data using REST API calls. Currently, there is no limit on the amount of time the user’s history data are stored in DASE and there is no user action involved in this step.

### AQI Calculation

#### General Equation

The Indoor Air Quality Index is calculated using the weighted mean formula. The contribution of each pollutant is multiplied by its weightage, whose calculation is explained in the next section, and divided by the sum of pollutant weightages.

This can be represented by the formula:



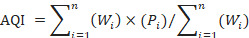



where *W_i_* is weightage of pollutant *i* and *P_i_* is the reading of pollutant *i*.

#### Calculation of Pollutant Weightages

Weightages for each of the pollutants have been calculated based on the concentrated means and their contribution to different AQI levels.

Individual AQIs (Table 2) and the breaking points for the concentration mean of different pollutants in a fixed cycle were used to arrive at the weights of each pollutant at the respective AQI levels. Weightage of each pollutant’s mean concentration at each AQI level was calculated by measuring the fractional contribution to the AQI.

**Table 2:** Individual Air Quality Indexes and the breaking points for the concentration mean of pollutants [49]^a,b^.

Indoor Air Quality Index	Sulfur dioxide 24 hours	Sulfur dioxide 1 hour^c^	Nitrogen dioxide 24 hours	Nitrogen dioxide 1 hour^c^	PM_10_^d^ 24 hours	Carbon monoxide 24 hours	Carbon monoxide 1 hour^c^	Ozone 1 hour	Ozone 8 hours	PM_2.5_^e^ 24 hours
0	0	0	0	0	0	0	0	0	0	0
50	50	150	40	100	50	2	5	160	100	35
100	150	500	80	200	150	4	10	200	160	75
150	475	650	180	700	250	14	35	300	215	115
200	800	800	280	1200	350	24	60	400	265	150
300	1600	^f^	565	2340	420	36	90	800	800	250
400	2100	^f^	750	3090	500	48	120	1000	^g^	350
500	2620	^f^	940	3840	600	60	150	1200	^g^	500

^a^Data presented are mean values.

^b^Sulfur dioxide (not collected by AMS) and nitrogen dioxide, which are primarily outside pollutants, are excluded from the indoor Air Quality Index calculation. Carbon dioxide and volatile organic compounds are much more common indoors and are more relevant and considered in the indoor Air Quality Index calculation.

^c^The concentration means of 1-hour sulfur dioxide, nitrogen dioxide, and carbon monoxide just adapt to the real-time calculation for Indoor Air Quality Index, but the concentration means of 24-hour sulfur dioxide, nitrogen dioxide, and carbon monoxide were used to calculate for a whole day.

^d^PM_10_: particulate matter with diameter of ≤10 μm.

^e^PM_2.5_: particulate matter with diameter of ≤2.5 μm.

^f^The concentration mean of 1-hour sulfur dioxide higher than 800 μg/m^3^ is calculated with the concentration mean of 24-hour sulfur dioxide.

^g^The concentration mean of 8-hour ozone higher than 800 μg/m^3^ is calculated with the concentration mean of 1-hour ozone.

#### tVOC Information

Values from Table 3 have been considered for the corresponding AQI windows, with the midpoint of concentration breaking points chosen as pollutant representation, which is used as the denominator, and the breaking point of the IAQI range as the numerator, similar to calculations performed for other pollutants.

**Table 3:** Individual AQIs and the breaking points for the concentration mean of VOCs and others [50].

Level	AQI^a^ range	VOC^b^ (µg/m^3^) concentration (BP_LO_–BP_HI_)^c^	CO^d^ (µg/m^3^) concentration (BP_LO_–BP_HI_)	PM^e^ (µg/m^3^) concentration (BP_LO_–BP_HI_)	Description
A	0-50	0-200	0-4.99	0-30	Good
B	51-100	201-350	5-9.99	31-90	Moderate
C	101-250	351-500	10-14.99	91-140	Unhealthy
D	251-400	501-757	15-2000	141-750	Very unhealthy

^a^AQI: Air Quality Index.

^b^VOC: volatile organic compound.

^c^BP: breaking point (LO: low; HI: high).

^d^CO: carbon monoxide.

^e^PM: particulate matter.

#### CO_2_ Information

Values from Table 4 have been considered for the corresponding AQI windows, with the midpoint of pollutant concentration calculated and chosen as pollutant representation, which is used as the denominator, and the breaking point of the IAQI range as the numerator, as is the case with others.

**Table 4:** Individual Air Quality Indexes and the pollutant concentration ranges of carbon dioxide and others [51].

Carbon monoxide (ppm)	Carbon dioxide (ppm)	Hydrogen (ppm)	Ammonia (ppm)	Ethanol (ppm)	Hydrogen sulfide (ppm)	Toluene (ppm)	Oxygen (%)	Indoor Air Quality Index	Health effects
0-0.2	0-379	0-1	0-24	0-0.49	0-0.00033	0-0.0247	20.95	0-50	Good
0.21-2	380-450	1.1-2	25-30	0.5-10	0.00034-1.5	0.0248-0.6	19-20.9	51-100	Moderate
2.1-9	451-1000	2.1-3	31-50	11-49	1.6-5	0.7-1.6	15-19	101-150	Unhealthy for sensitive individuals
9.1-15.4	1001-5000	3.1-5	51-100	50-100	6-20	1.7-9.8	12-15	151-200	Unhealthy
15.5-30.4	5001-30,000	5.1-8	101-400	101-700	21-50	9.9-12.2	10-12	201-300	Very unhealthy
30.5-50.4	30,001-40,000	8.1-10	401-500	701-1000	51-100	12.3-100	<10	301-400	Hazardous

The final weightage factor of each pollutant was calculated by taking the arithmetic mean as shown in Tables 5-10.

#### Pollutant Weightage Formula



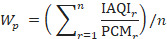



where *W_p_* is the weightage of pollutant; IQAI*_r_* is the individual AQI pollution level; and PCM*_r_* is the corresponding concentration threshold mean of a pollutant.

#### Weightage Worksheet Tables by Pollutant

**Table 5:** Weightage calculation for PM_10_^a^.

Indoor Air Quality Index	PM_10_ 24-hour mean	PM_10_ weightage
50	50	1
100	150	0.666666667
150	250	0.6
200	350	0.571428571
300	420	0.714285714
400	500	0.8
500	600	0.833333333
Final weightage		0.740816327

^a^PM_10_: particulate matter with diameter of ≤10 μm.

**Table 6:** Weightage calculation for CO^a^.

Indoor Air Quality Index	CO 1-hour mean	CO weightage
50	5	10
100	10	10
150	35	4.28571429
200	60	3.33333333
300	90	3.33333333
400	120	3.33333333
500	150	3.33333333
Final weightage		5.37414966

^a^CO: carbon monoxide.

**Table 7:** Weightage calculation for O_3_^a^.

Indoor Air Quality Index	O_3_ 1-hour mean	O_3_ weightage
50	160	0.3125
100	200	0.5
150	300	0.5
200	400	0.5
300	800	0.375
400	1000	0.4
500	1200	0.416666667
Final weightage		0.429166667

^a^O_3_: ozone.

**Table 8:** Weightage calculation for PM_2.5_^a^.

Indoor Air Quality Index	PM_2.5_ 24-hour mean	PM_2.5_ weightage
50	35	1.428571429
100	75	1.333333333
150	115	1.304347826
200	150	1.333333333
300	250	1.2
400	350	1.142857143
500	500	1
Final weightage		1.248920438

^a^PM_2.5_: particulate matter with diameter of ≤2.5 μm.

**Table 9:** Weightage calculation for tVOC^a^.

Indoor Air Quality Index	tVOC mean	tVOC weightage
50	100	0.5
100	275	0.363636364
250	425	0.588235294
400	628	0.636942675
Final weightage		0.522203583

^a^tVOC: total volatile organic compound.

**Table 10:** Weightage calculation for CO_2_^a^.

Indoor Air Quality Index	CO_2_ mean	CO_2_ weightage
50	341	0.146627566
100	747	0.133868809
150	1305	0.114942529
200	5400	0.037037037
300	31,500	0.00952381
400	63,000	0.006349206
Final weightage		0.074724826

^a^CO_2_: carbon dioxide.

### Data Analysis

**Figure 8:**
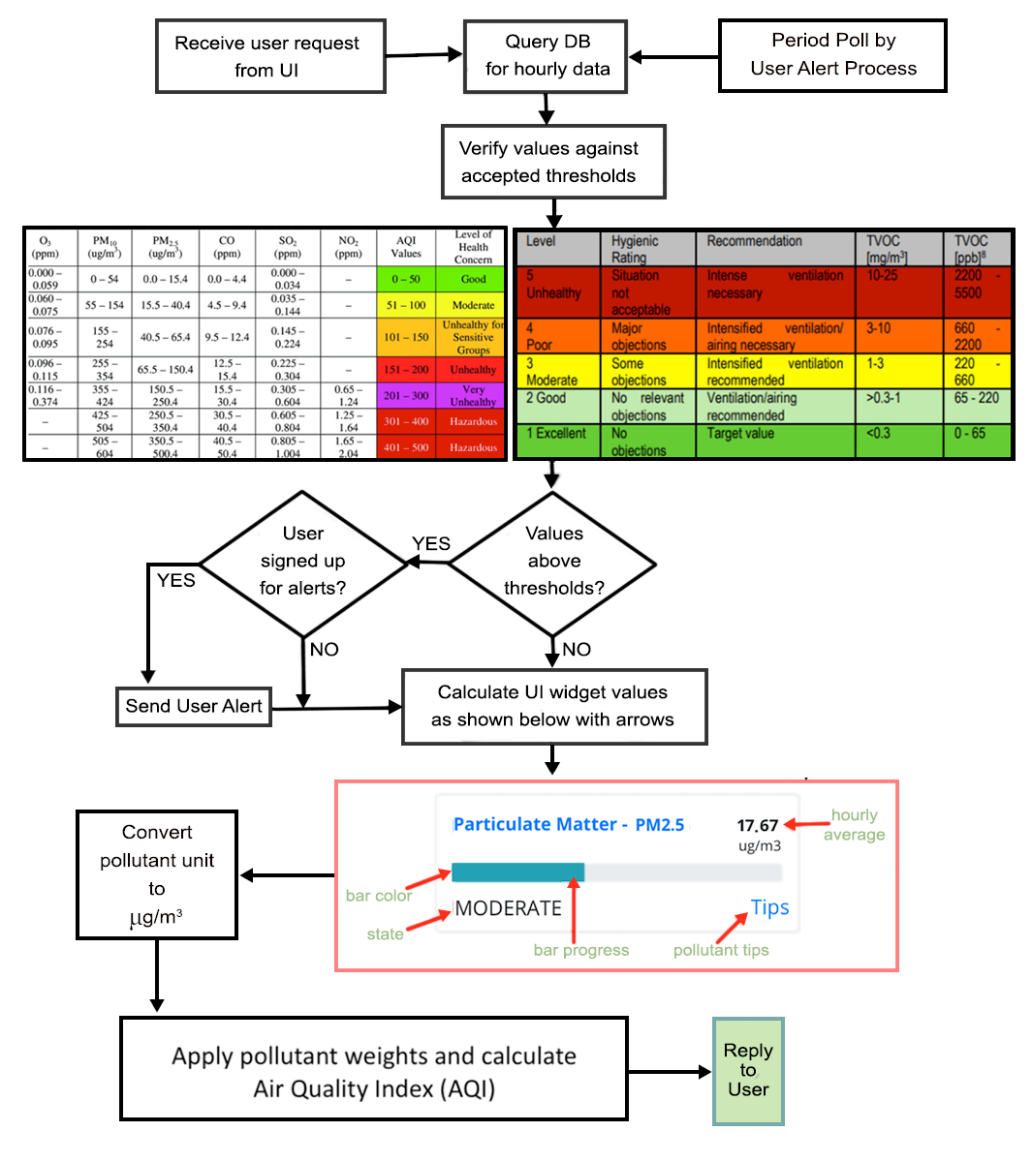
DASE data flow on the web server used to populate the Atmosome UI or send a user alert. DASE: data analysis and storage engine; DB: database; UI: user interface.

The data analysis flowchart is depicted in Figure 8. Analysis of the collected data from the DCS is automatically executed in the background and is transparent to the user. Upon receiving a request from the UI, or through the background user alert process, the database is queried to retrieve data. The data are validated against thresholds predetermined by environmental safety limits [52,53]. If the user is registered to receive alerts and the values exceed safe thresholds, an alert is sent to the user. Additionally, the values and corresponding qualitative metrics of each pollutant are determined. Finally, AQI is computed and the data are returned.

The authors considered including an adaptive threshold for each analyte to account for spatial, temporal, and environmental variations. However, AMS is recalibrated in each new usage location and is intended for indoor use. Thus, environmental variations are small and no substantial error between AMS and COTS sensors was observed when analyzing the data. Nonetheless, it is well-known that MQ sensors exhibit high temperature coefficients and sensitivity to humidity [54]. Drift of these sensors is low and ongoing recalibration every 3 months is more than sufficient to address such drift [54]. Considering these factors, the ranges at which the AQI considered are absolute, but the unit itself is adjusted to account for location. Further, the sample rate is considered sufficient for the application. In the data that follow, it does not appear that the sensors are undersampled, so temporal variation is not considered in this embodiment. Nonetheless, one of the goals for the future work is to automate the calibration process and also allow customization of the calibration interval according to the user’s choice.

### Data Alerts

If a user opts to be alerted when pollutants are above the optimal threshold, DASE sends an email and SMS text message alerts. This could be invaluable in preventing accidents or calling for emergency services in the case of an NG leakage or similar emergency.

### Data Display

#### Current UI Module and Plans for the Next Version

The UI module displays the air quality information to users. The user accesses the UI at the atmosome website [55]. There is a dropdown menu to select the user’s zip code.

The next version of the atmosome UI will include a login screen for the user, instead of the current zip code selection. The various parts of the UI displayed to the user are described below.

#### Hourly Dashboard

**Figure 9:**
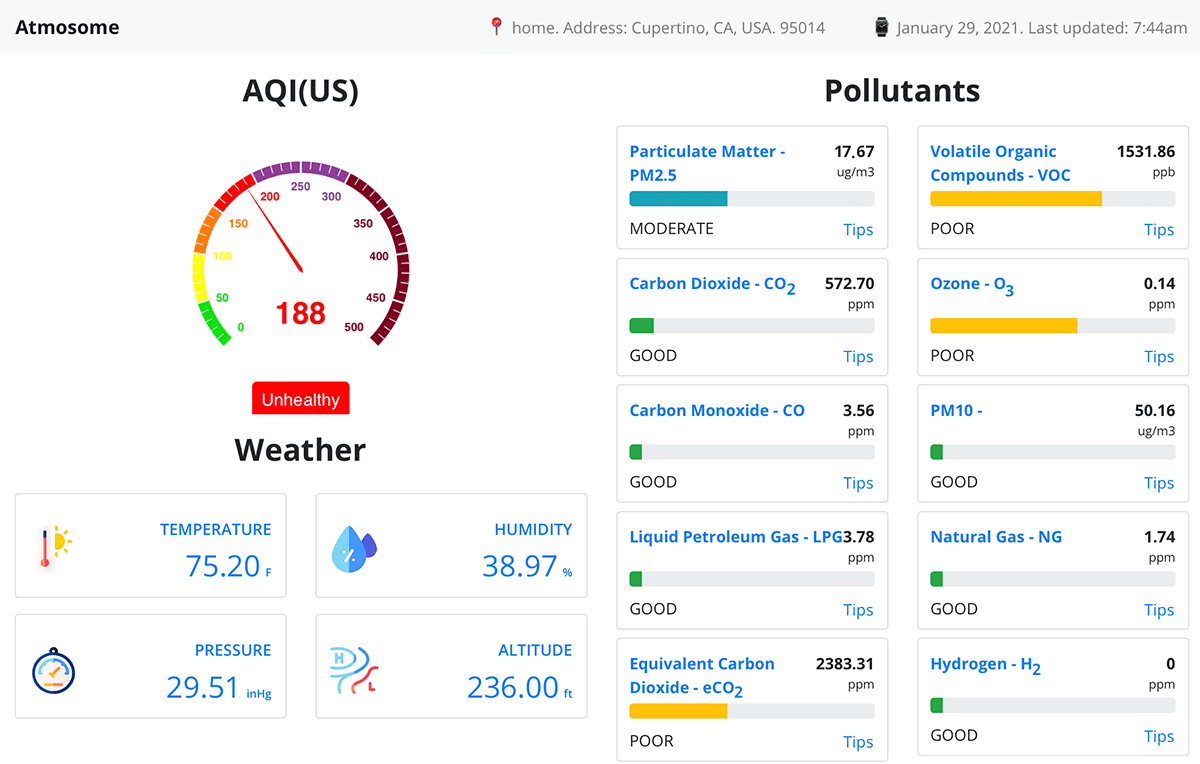
Representative example of hourly dashboard of user’s atmosome data displayed by AMS UI. The values of 4 environmental parameters are also quantitatively presented below the AQI gauge. AMS: Atmosome Measurement System; AQI: Air Quality Index; PM_10_: particulate matter with diameter of 10 μm or less; UI: user interface.

The dashboard (Figure 9) presents the user with an “at-a-glance” state of their indoor air quality using a gauge that reflects AQI. The gauge conforms to the conventions of the US AQI gauge. The AQI is computed based on the pollutants that show the most variability due to user lifestyle, including PM_2.5_, tVOC, CO_2_, O_3_, CO, and PM_10_. The past hourly averages sample of 10 pollutant analytes is presented visually and quantitatively. Thresholds for each analyte are predetermined by environmental safety limits. Relative assessments of levels are color coded and reported as GOOD, MODERATE, POOR, or BAD. Four environmental parameters, including temperature, pressure, humidity, and altitude, are also shown quantitatively.

#### Recommendations

**Figure 10:**
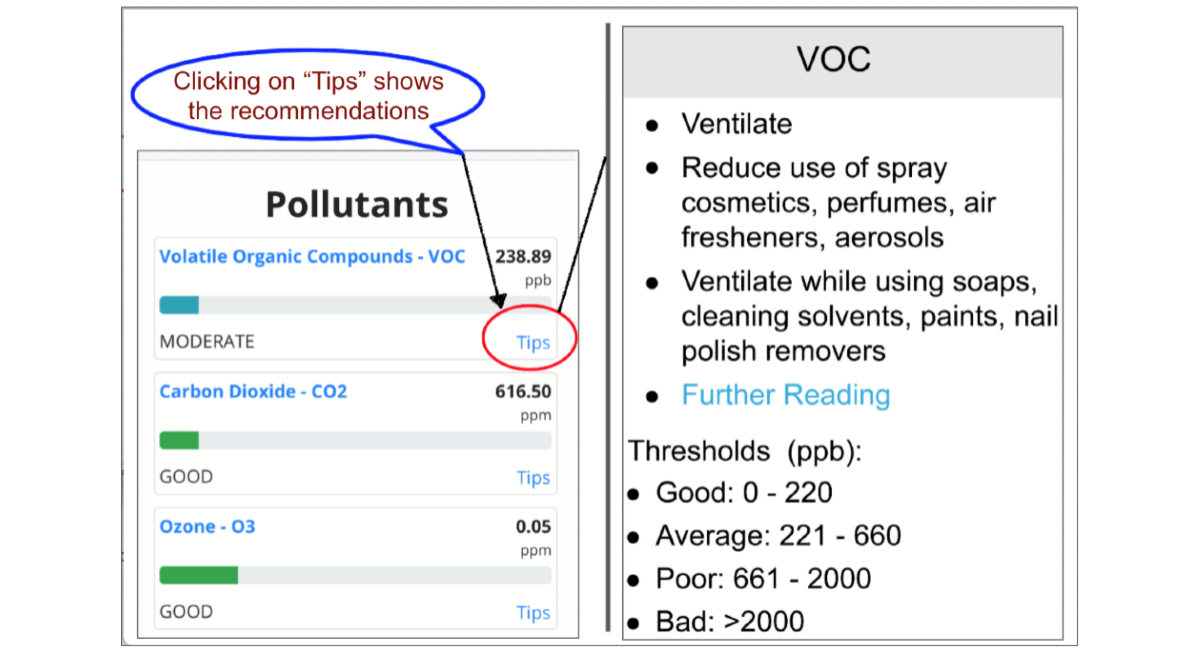
Lifestyle recommendations to improve atmosome quality, provided to the user in AMS UI. Clicking on the “Tips" link of each of the pollutants in the UI dashboard, as shown, opens up a popup box showing the quantitative thresholds associated with the pollutant and gives the user recommendations on managing it to maintain healthy levels. AMS: Atmosome Measurement System; ppb: parts per billion; UI: user interface; VOC: volatile organic compound.

**Figure 11:**
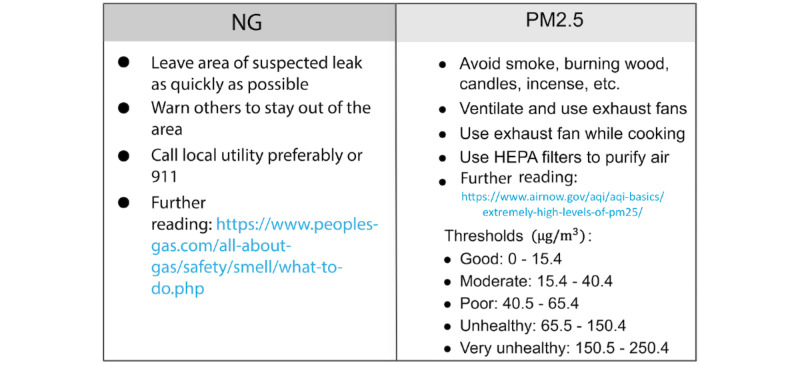
Examples in the AMS UI that show the recommendations and thresholds for NG and PM2.5. AMS: Atmosome Measurement System; NG: natural gas; PM_2.5_: particulate matter with diameter of 2.5 μm or less; UI: user interface.

Each pollutant in the dashboard is associated with detailed information about acceptable thresholds and specific suggestions on how to manage it to be within healthy limits. This is shown in Figures 10 and 11.

#### Average Exposure

**Figure 12:**
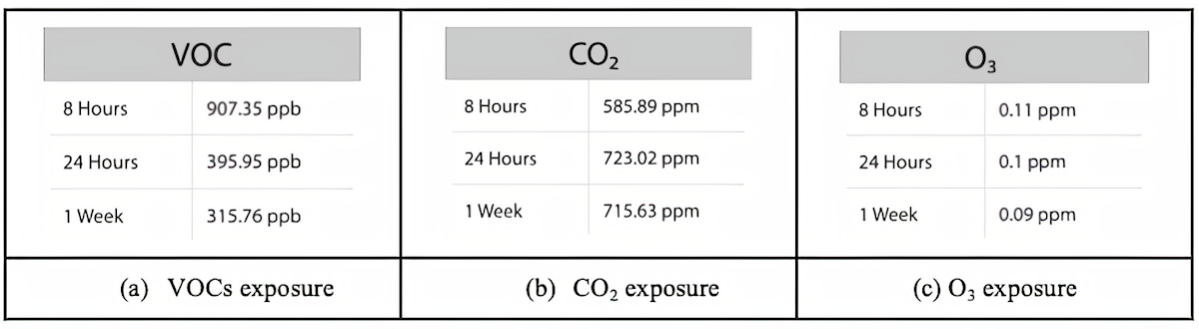
An example of AMS UI showing the average exposure statistics for different pollutants across various periods, ranging from 8 hours to 1 week. AMS: Atmosome Measurement System; CO_2_: carbon dioxide; O_3_: ozone; ppb: parts per billion; ppm: parts per million; UI: user interface; VOC: volatile organic compound.

The cumulative average values of different pollutants indicate the overall exposure across various periods as shown in Figure 12. These data provide potential insights into the underlying causes of poor AQI, which could be correlated with specific living conditions.

#### Quarterly Temporal Graphs

**Figure 13:**
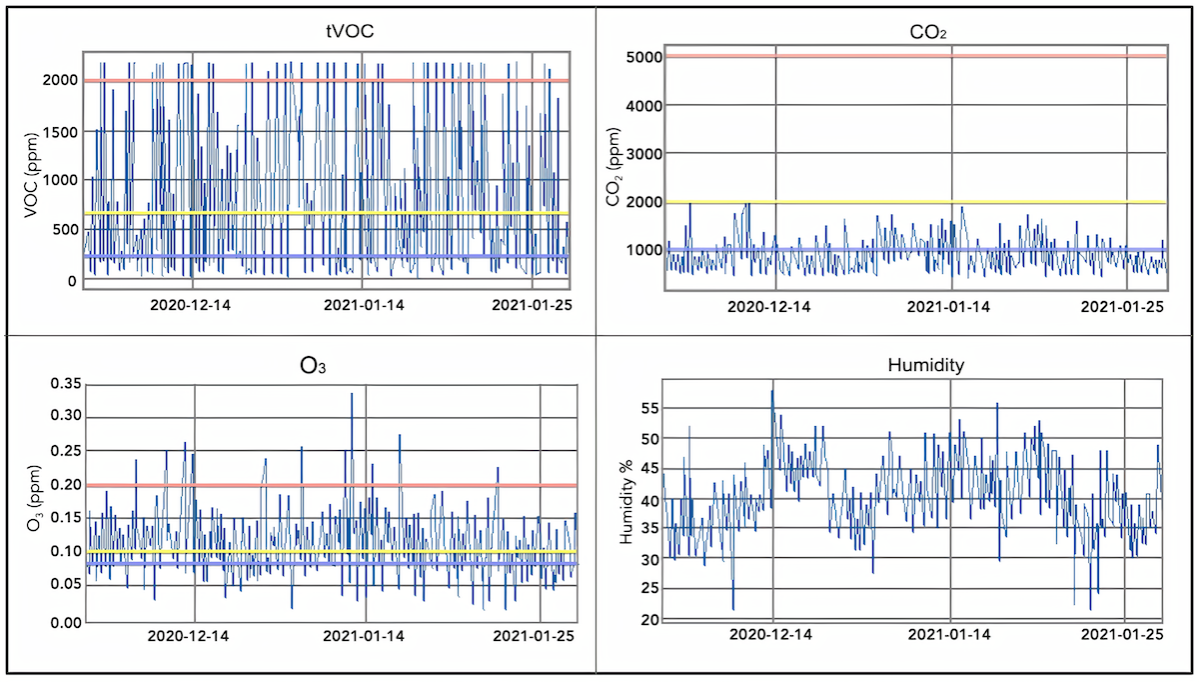
An example of AMS UI showing graphs of the user’s quarterly trends of tVOC, CO_2_, O_3_, and humidity data streams. AMS: Atmosome Measurement System; CO_2_: carbon dioxide; O_3_: ozone; tVOC: total volatile organic compound; UI: user interface.

The UI time-series data display quarterly data from the different pollutant data streams (Figure 13). This enables users to visualize trends over time and gain deeper insights into which pollutants are affecting their air quality most substantially. For example, tVOC measurements are noted to be highly variable due to indoor sources resulting from occupant lifestyle, including exposure to cosmetics, cleaning products, room refreshers, cooking fumes, and more.

#### Pollutants Information Page

**Figure 14:**
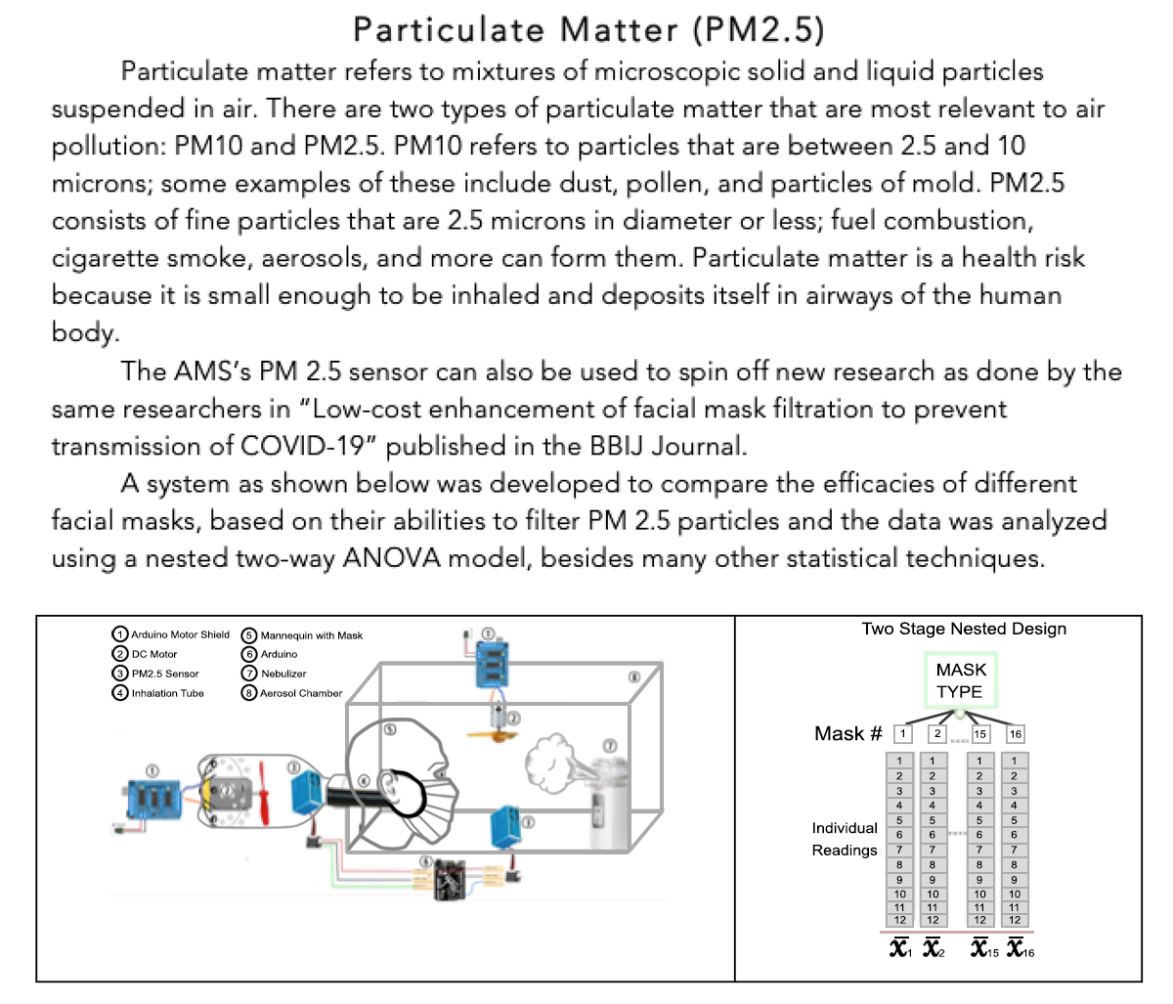
An example of AMS UI individual pollutant page that is displayed on a separate web page for every pollutant and can be found by selecting the pollutant name in the dashboard. These pages provide the user with greater detail about each pollutant. This includes information such as more details about the pollutant, common sources of the pollutant, possible research and data analysis that have been/could be done on the pollutant using the corresponding AMS sensor, and more. This figure is an example of the PM2.5 pollutant page. Besides providing more details about PM_2.5_, it shows how the AMS PM_2.5_ sensor and extensive statistical analysis of its data was used for new, internationally published research on low-cost enhancement of facial mask filtration. AMS: Atmosome Measurement System; PM_2.5_: particulate matter with diameter of 2.5 μm or less; UI: user interface.

For each category of pollutant, upon selecting its name in the UI, the user is taken to a new page that contains a brief description of the pollutant; associated health risks at different concentrations; and examples of how such data can be collected, graphed, and studied further are shown. This provides users further insights into each of the pollutants and the possibility of enabling additional research. An example description page is shown in Figure 14 for PM_2.5_.

#### REST API

An extended REST API is also available for advanced users and developers interested in conducting further research or data analysis using the DCS measurements. The REST API enables users to download their data in a .csv format*.* These data can be mined to gain deep insights into the dynamics of the various indoor air pollutants across time, address extreme or alarming conditions by taking appropriate corrective actions, and exploring possible connections between air pollution and various health conditions.

## Results

### Study Purpose

The purpose of this work was to measure various pollutants and other air quality metrics that affect individual environmental atmosomes. The following section presents the results of a variety of air quality metrics in selected environments and relates them with the conditions in their atmosome.

### Temperature and Humidity

**Figure 15:**
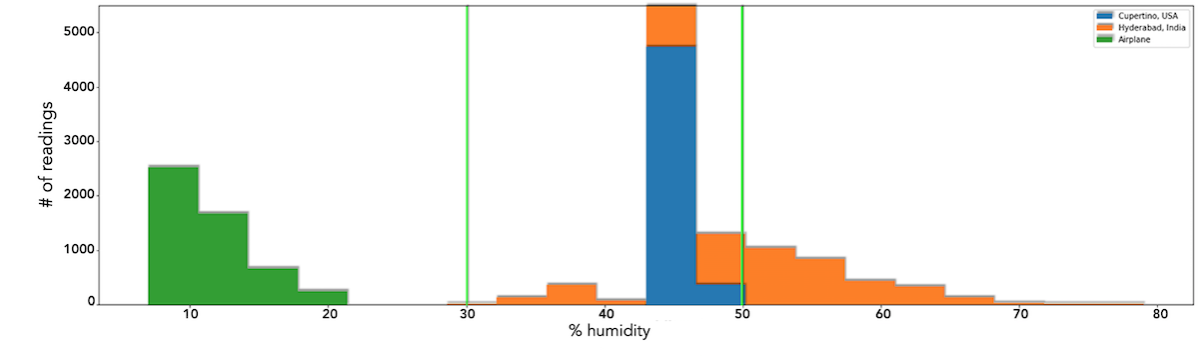
Relative Humidity (%): 30-50 marks the ideal range. Conditions within the airplane journey were low in humidity and conditions in Hyderabad went above the recommended range at times. The Cupertino home had ideal humidity values.

**Figure 16:**
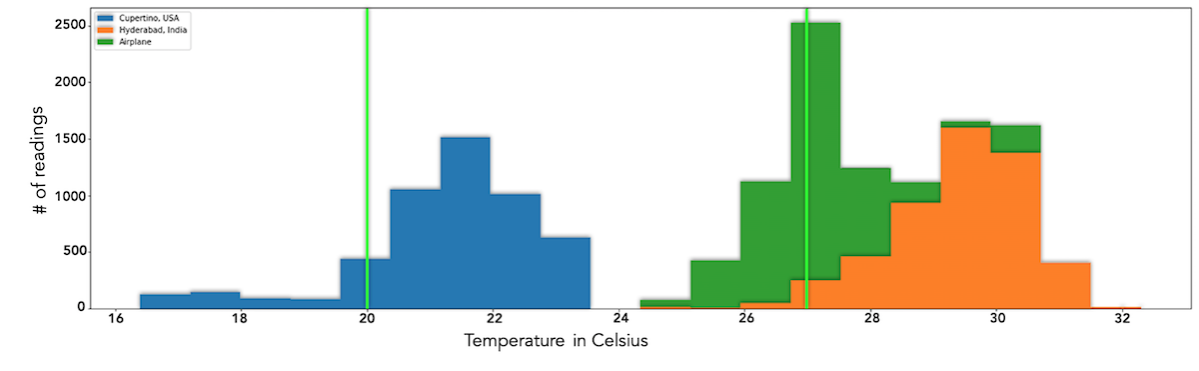
Temperature (°C): 20-27 marks a comfortable and healthy range. Conditions within the airplane journey and in Hyderabad were high in temperature. The Cupertino home had mostly ideal temperature values.

Figures 15 and 16 show the quantity and value of readings of relative humidity and temperature, respectively, taken at 3 different indoor locations: a home in Cupertino, California (shown in blue); a home in Hyderabad, Telangana, India (shown in orange); and an airplane economy cabin during a nonstop flight of 17 hours (shown in green). The low humidity readings from the airplane correlate with the dryness and discomfort often experienced by airplane passengers and align with the United States Centers for Disease Control and Prevention’s (CDC) air travel yellow book [56].

### Carbon Dioxide

**Figure 17:**
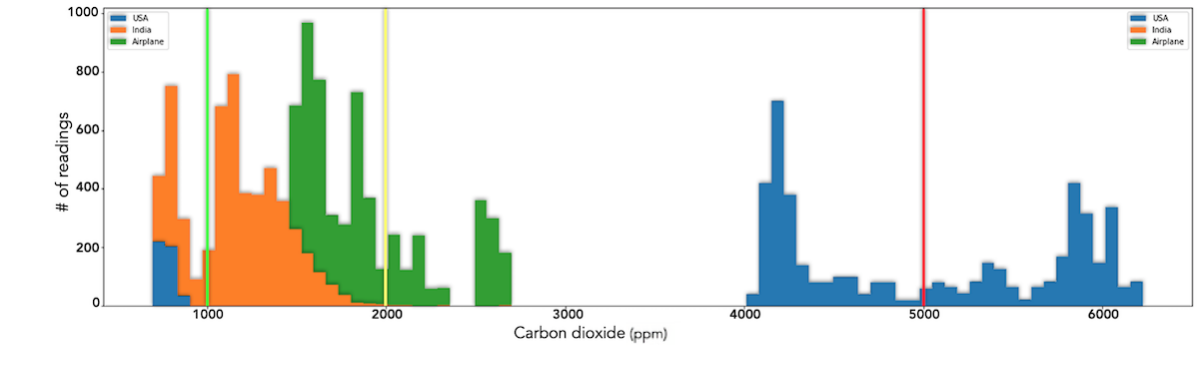
CO_2_ (ppm): 250-1000 is the safe range for typical indoor spaces with good air ventilation. Higher than 1000 leads to a range of adverse effects from drowsiness to headaches, nausea, and increased heart rate among other conditions. Conditions within the airplane journey and in Hyderabad were mostly ideal to moderate, while conditions in the Cupertino home were outside healthy limits. CO_2_: carbon dioxide; ppm: parts per million.

**Figure 18:**
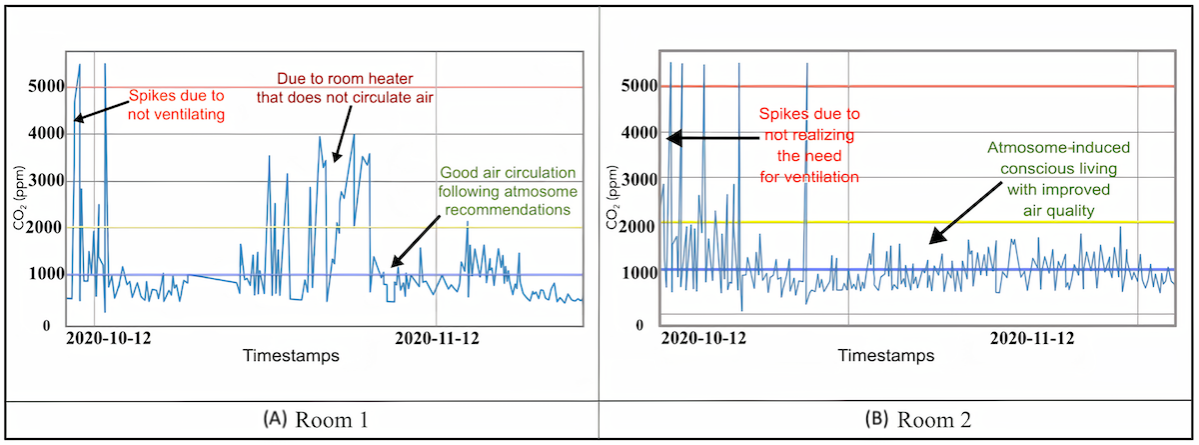
Temporal data for CO_2_ pollution in 2 different rooms in the Cupertino house: (A) Room 1; (B) Room 2. In both rooms, air conditions were initially unhealthy and then improved drastically. In Room 1, conditions became unhealthy again after some hours before improving once more. CO_2_: carbon dioxide.

Figure 17 shows a graph of CO_2_ measurements taken in the same environments as above. The readings in the home in the United States showed much higher indoor CO_2_ levels than the readings in a more polluted area in India. Further analysis has revealed, however, that the closed windows and doors throughout the day during winter in the United States reduced ventilation and increased CO_2_ concentration. Studies show that higher CO_2_ exposure can cause drowsiness [57]. These data highlight the importance of ventilation during the winter. The readings on the lengthy airplane journey confirmed that the DCS readings and published values by the airline were within the range of each other. Figure 18 illustrates CO_2_ readings in 2 rooms and shows the role AMS had in enabling the researcher to take corrective actions to improve indoor air quality.

### Particulate Matter 2.5 µm (PM_2.5_)

**Figure 19:**
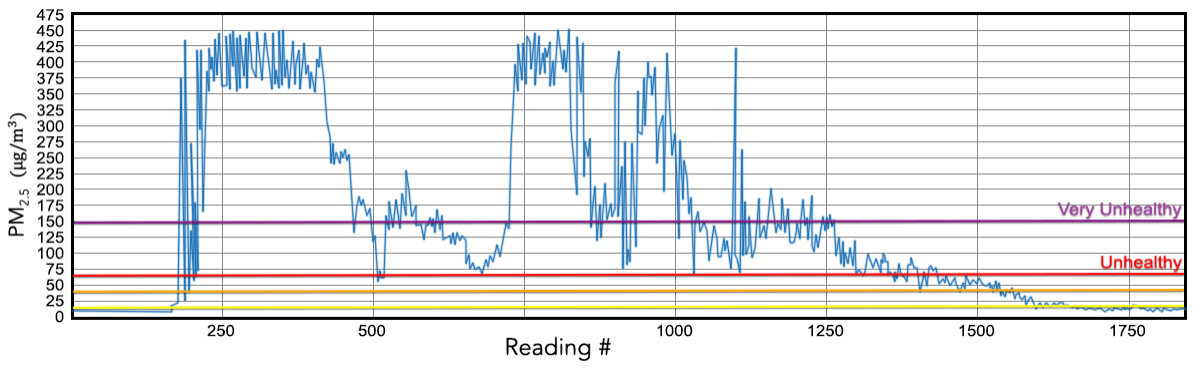
PM_2.5_ (µg/m3) readings in the kitchen while cooking food. Much of the time spent cooking was in very unhealthy air conditions. PM_2.5_: particulate matter with diameter of 2.5 μm or less.

Figure 19 depicts the variation in PM_2.5_ during the course of a typical home activity (cooking food). The onset of cooking is associated with a sharp, transient increase in the levels of PM_2.5_ followed by a sustained period of unhealthy PM_2.5_ levels. In this particular scenario, the user responded to the elevated concentration of PM_2.5_ by activating the exhaust fan, deactivating the stove, and opening all windows for improved cross-ventilation.

This set of mitigating actions lowered the PM_2.5_ levels but unhealthy concentrations (defined by the horizontal orange and purple lines in Figure 19) persisted even after cooking ceased. These observational data highlight the power and utility of AMS in identifying and mitigating substantially unhealthy levels of indoor air quality. A longitudinal analysis of such data has the potential to offer rich insights into the interaction between indoor air pollutants and respiratory health outcomes, and aid data-driven health policy research.

### Volatile Organic Compounds and Ozone

**Figure 20:**
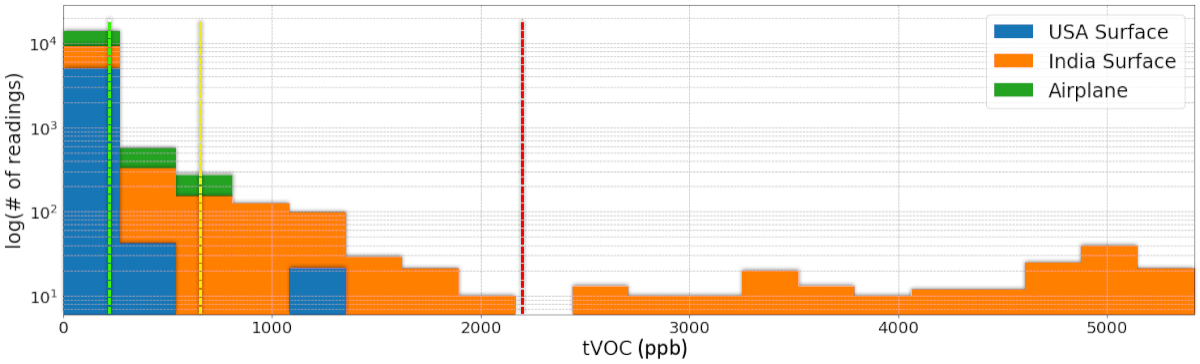
tVOCs (ppb): 0-200 is the safe range. Values above 2200 ppb are extremely unhealthy. Conditions in the airplane journey and in the Cupertino home were typically ideal, whereas conditions in Hyderabad were often very unhealthy. ppb: parts per billion; tVOC: total volatile organic compound.

Raw data stored in DASE were downloaded in .csv format using its REST API for advanced users. The data were graphed in a python notebook using matplotlib. As shown in Figure 20, there is a substantial difference between Indian and American household air pollution levels in terms of tVOC measurements. The difference in results could be attributed partly to the substantially higher levels of air pollution in India compared with those in the United States. The current results are consistent with previous results suggesting vehicle exhaust as one of the leading sources of VOC-related pollution in India [58].

**Figure 21:**
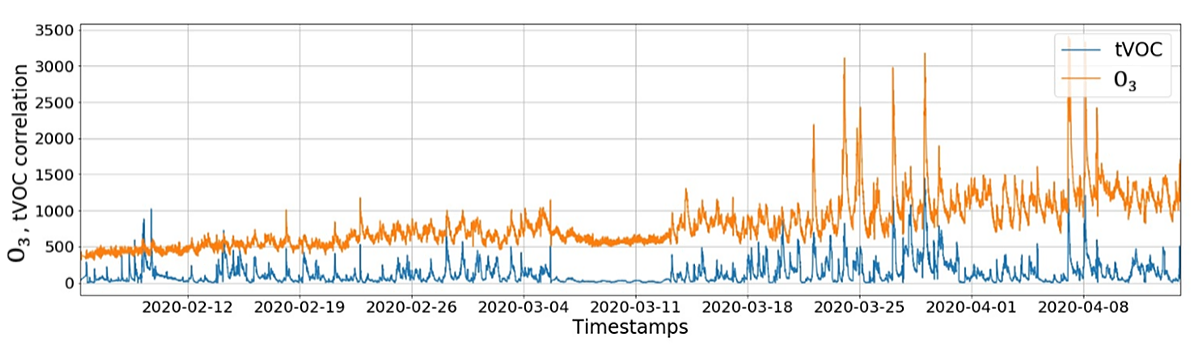
Ozone (ppb): Ozone levels from February to April 2020 at a home in the Sierra Mountains, El Dorado County, CA, USA. O_3_: ozone; ppb: parts per billion; tVOC: total volatile organic compound.

Figure 21 displays a strong correlation between tVOC and ground-level O_3_ measurements. For the most part, spikes in tVOC result in increases in O_3_ levels that cause nitrogen oxides to react with tVOC in the presence of sunlight to create ground-level O_3_.

**Figure 22:**
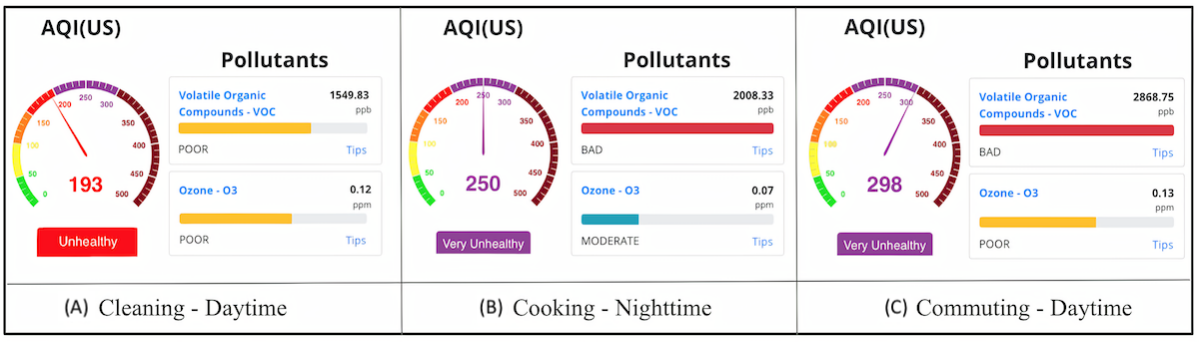
tVOC readings during (A) home cleaning, (B) cooking, and (C) commuting by car. These statistics are displayed on AMS user interface. AMS: Atmosome Measurement System; tVOC: total volatile organic compound.

Figure 22 displays tVOC readings recorded during various daily activities such as house cleaning, cooking at night, and commuting by car. The tVOC values were 1549, 2008, and 2868 ppb, respectively, for the aforesaid activities, well above the accepted moderate range of 220-660 ppb (presented in Figure 8), and demonstrate the high levels of tVOC during innocuous common activities [59]. The results also show that tVOC readings impact the indoor AQI value.

**Figure 23:**
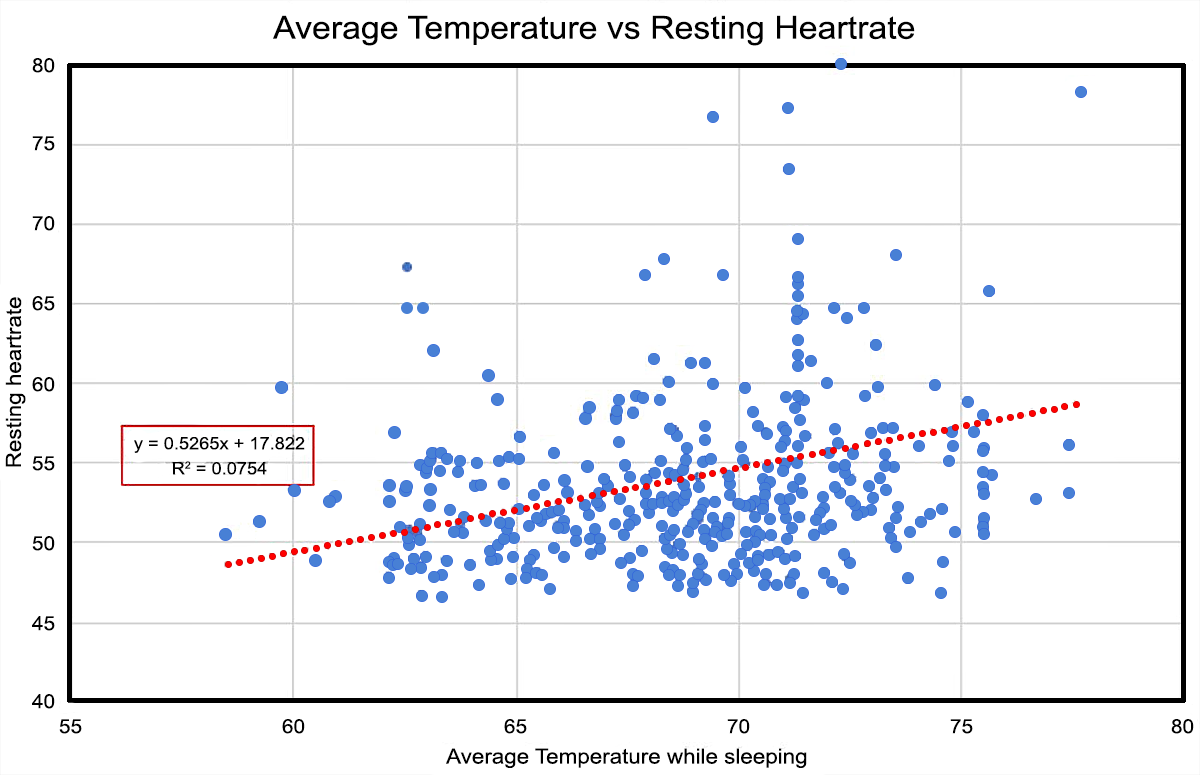
Personal atmosome biological modeling. This scatterplot and line-of-best-fit display how ambient temperature affects resting heart rate reflected by data from the GoldenCheetah OpenData Project.

Studies carried out in 2003 by the California Air Resources Board show that cleaning products alone account for the release of 7.4 t of VOCs per day and that their various health effects include asthma attacks and eczema [60]. AMS can furnish necessary information to take precautions in such situations (eg, a spike in tVOC levels). Homes with infants or expecting parents often undergo more cleaning than typical. AMS, however, suggests that a presumably “well-cleaned” house may, in fact, pose a heightened risk of childhood asthma to infants [60], delays in child language development [61], or prenatal exposure to the fetuses that may impact their postnatal growth [62].

In summary, these results show that IAQ may be at unhealthy levels while conducting typical daily activities. AMS is able to track air quality through its sensors and indicate the impact of harmful levels of indoor air pollutants such as O_3_, tVOC, CO_2_ levels, and the presence of PM_2.5_ inside homes.

## Discussion

### Principal Findings

This work has aimed to determine the performance of a low-cost AMS in indoor spaces in terms of gathering sensor data for a variety of air pollutants, transferring data reliably to an analysis engine in the cloud, displaying air quality monitoring results to the user, and sending alerts when the pollutants exceed safe thresholds. This system also provided a way for users to choose to share their IAQ data anonymously for further health research, and for users and researchers to retrieve data using REST APIs for further analysis and data analysis. This can be a foundation for building a public IAQ database across geographical regions.

The results establish that AMS is effective in analyzing multipollutant data streams in multiple settings, displaying AQI value, hourly visual and quantitative data of 18 pollutants, average exposure statistics of various pollutants, and the user’s quarterly pollutant exposure graphs. The dashboard also includes tips for each pollutant that display the GOOD, MODERATE, or POOR thresholds, enabling the user to better interpret the values and graphs shown in the UI and receive recommendations for keeping various pollutants in check with simple mitigation. The individual pollutant pages give the user further insights into the pollutant and show novel research in which AMS sensors and data collection have been employed. The system is also effective in sending alerts when pollutants exceed safe thresholds. The following sections discuss the findings of the work, which are corroborated by previous work and studies of a similar nature.

### Particulate Matter

Particulate matter refers to mixtures of microscopic solid and liquid particles suspended in the air. There are 2 types of particulate matter that are most relevant to air pollution: PM_10_ and PM_2.5_. PM_10_ consists of particles that are between 2.5 and 10 μm (diameter); some examples of these include dust, pollen, and particles of mold. PM_2.5_ consists of fine particles that are 2.5 μm in diameter or less; some examples of these include fuel combustion, cigarette smoke, and aerosols. Particulate matter is a health risk because it is small enough to be inhaled and deposits itself in the airways of the human respiratory system. Smaller particles can lodge themselves deep in the lungs or enter the bloodstream. Even short-term exposure to PM_2.5_ has been associated with worsening respiratory diseases and can lead to emergency care. Long-term exposure (ie, months to years) has been linked to premature death, especially in people with chronic conditions, and leads to reduced lung function in children [63]. The EPA set the maximum 24-hour exposure limit to PM_2.5_ to 35 µg/m^3^ and annual exposure limit to 12 µg/m^3^ [64].

Quite interestingly, results of this work clearly indicate that when cooking is involved, the level of PM_2.5_ increases well beyond recommended exposure limits and these levels remain high even after cooking has ceased. While it was observed that ventilation could mitigate levels to some extent, it is imperative that cooking methodologies are modified to reduce the emission of PM_2.5_. A study that focused on emission of PM_2.5_ from cooking in homes found that the levels were raised 20-40-fold to 160 µg/m^3^ in the kitchen and 10-fold in the nearby living room to 60 µg/m^3^ [65], clearly corroborating the results of this work. Measuring particulate matter in indoor air can direct researchers toward finding easily implementable corrective actions to reduce exposure levels.

### Carbon Dioxide

CO_2_ is considered to be a dominant air pollutant. Moderately high concentrations of CO_2_ in indoor air can lead to drowsiness, fatigue, and headaches. Increasing amounts can cause dizziness and nausea [66]. In the experimental data, CO_2_ levels increased dramatically in poorly ventilated rooms as shown in the temporal data in Figure 18. In both the rooms, air conditions were initially unhealthy and then improved drastically. In room 1, conditions became unhealthy again after some hours due to a room heater that did not circulate air. Taking advice furnished by AMS and adding a fan, along with regular ventilation, improved conditions again. These results corroborate the findings of a previous work in the Texas elementary schools that monitored CO_2_ levels in 120 randomly selected classrooms in 2 school districts [67]. The simple process of improving ventilation and air circulation can reduce high concentrations of CO_2_ and improve comfort.

### Volatile Organic Compounds

The experimental results show that even seemingly routine daily activities can induce significant tVOC release. A variety of household items, such as candles, cooking fumes, room fresheners, cosmetics, cleaning products, and paints, emit VOCs. These are organic chemicals that are usually in gaseous form at room temperature and are photo-chemically active. Short-term exposure to VOCs can cause optic or respiratory irritation, headaches, memory lapses, and dizziness. Long-term exposure can cause nausea, fatigue, organ damage, and cancer [68]. Results shown in Figure 22 based on an AMS report of activities, such as cleaning, cooking, and commuting, corroborate the findings of previous work [69-72].

### Ozone

Readings taken while cleaning, cooking, and commuting, as shown in Figure 22, highlighted a correlation between activities that lead to elevated VOC levels in the presence of sunlight and elevated O_3_ levels. In support, studies in Los Angeles have found as many VOCs being emitted from household products as from vehicle exhaust pipes, which then react in the presence of sunlight to produce ground-level O_3_ [73]. These results match the EPA’s report that mentions the adverse effects of synthetic chemicals and emissions caused by cars, power plants, and other industrial setups that react in the presence of sunlight to increase ozone levels [74]. The results of this work show that the same levels of VOCs do not elevate O_3_ as much at night, as there is no sunlight to facilitate such a chemical reaction.

Ground-level O_3_ can trigger a variety of health problems, including chest pain, coughing, throat irritation, and airway inflammation. It can also worsen bronchitis, emphysema, and asthma [74]. This can lead to the need for increased medical care [75]. In 2016, 90% of noncompliance to the national ambient air quality standards in the United States was due to O_3_. Both short- and long-term exposures to O_3_ at concentrations below the current regulatory standards are associated with increased mortality from respiratory and cardiovascular diseases [76].

### Temperature

The DCS can measure both events induced by high temperature (eg, the formation of ground-level O_3_ and low-temperature triggers [77]) and brown adipose tissue metabolism [78]. The direct impact of temperature reflected in a user’s heart rate data stream during their sleep was characterized. An increasing trend in ambient temperature even in the 68-80°F (20-27°C) range and most definitely beyond (>27°C; Figure 16) could be unhealthy. This observation is supported by a previous study highlighted in Figure 23, which shows data collected for 1 user over 424 nights using an iPhone with the Sleep Cycle app and a Garmin Fenix 5 Watch [79]. Clear increases in the user’s circulation flow (ie, reflected by resting heart rate) were observed and appear to be correlated with increases in ambient temperature.

### Humidity

Lower humidity levels can cause skin dryness, corneal dryness, dry nasal passages, and sinusitis [80], while higher relative humidity levels can promote the growth of mold, bacteria, and viruses. According to previous studies, low humidity levels lead to increased aqueous tear evaporation [81,82]. The low humidity values in the flight shown in Figure 15 corroborated with the researcher’s discomfort due to dry eye symptoms during the flight.

### Significance

Previous studies have shown that several air pollutants, including PM_2.5_, PM_10,_ tVOC, CO, CO_2_, and more, can be present at much higher quantities in indoor air than in the outdoors [83]. There has been an increasing concern among the scientific community about the connection between IAQ and the impact of personal health [84].

The study results are significant in several ways. Data access in AMS is both simple and comprehensive. Real-time AMS data can be obtained and analysis performed from any device through a progressive web application. The website also includes historic data logs and recommendations on how to improve the user’s current air quality. Because it uses a cloud-based API, it opens the possibility to integrate new types of analytical graphs or recommendations into the system without the users needing to update their device. With all the air quality measurements collected and posted in tandem, there is an increased knowledge about the interconnections and impacts of pollutants on an individual, community, or specific demographic. Other researchers can also tap into the system to download data and include their own analyses for their unique studies.

### Limitations

Because the DCS unit’s calibration is not performed on a large scale, the calibration training set is limited when developing data correction algorithms. Another limitation is that the data were collected only by the researcher, and not external participants, which indicates that the data set is small and does not provide insight into various indoor pollutants affecting various living situations. This limits the development of training sets for learning algorithms to generate optimal individual weights for each of the pollutants to calculate AQI and to generate customized optimal recommendations for managing the pollutants to maintain healthy indoor air quality. While the DCS unit can be USB powered and portable when providing 13 data streams, due to increased power consumption for the comprehensive 22 data streams model, it requires to be plugged into a wall socket, thereby limiting its portability.

### Future Work

Future research could implement AMS on a large scale to safeguard public and personal health. Users can anonymously share their data to help researchers draw connections between indoor air pollution and public health and to further research in the development of new modeling techniques for public health, low-cost sensor data correction algorithms, and sensor modeling. The larger vision of AMS is to build a public database for indoor air, like EPA’s Air Quality System database [11,12] for ambient air. The REST APIs in the system can be used to access the open database populated by users willing to share their data.

AMS could include dynamic recalibration of the devices installed at various settings over the internet. With this auto-calibration, sensors would maintain the highest accuracy possible and users would not have to perform manual calibration themselves.

AMS can be researched for reduction of power consumption, while still providing the comprehensive set of all 22 data streams, thus maximizing its portability.

AMS can be used within households to compare indoor air quality levels between neighbors, or on a grander scale within communities. Large-scale distribution of AMS can lead to an expansion of preventative health approaches from an individual level to a community level and can provide valuable insights into public health.

Other possible enhancements include making the high-power model portable, and adding physical indicators, such as sound and light on AMS hardware, which can help indicate the hazardous levels of pollutants in a more noticeable way.

Cybernetic and navigational health approaches enable individuals to be in control of their health throughout their lives so that they have the necessary information to always maintain an ideal state of health [85,86]. Continued research in this field will focus on expanding into other health domains, improving quality metrics, and developing methods to combine atmosome data with other data streams to provide uniquely tailored lifestyle recommendations.

Last, AMS could implement more sophisticated software algorithms, such as an artificial neural network, as demonstrated in [27], and machine learning, as demonstrated in [30], and adaptive thresholding. In particular, the simplest algorithm to implement would be to account for environmental variation (eg, temperature and humidity) using an adaptive threshold to improve sensor accuracy. Specifically, and referring to [54], an adaptive threshold or the computed gas concentration could be computed as a function of temperature and humidity. This would significantly increase the sensitivity of the MQ sensors. Other algorithms likely exist and could be explored with the intention of maintaining a low-cost and small sensor solution as opposed to transitioning to an alternative sensor.

### Conclusions

In this work, the concept of an atmospheric exposome (atmosome) was presented and a low-cost approach to leverage multimodal sensors and cloud data storage in building a personal atmosome was proposed. This work shows that AMS offers both the concept and system to quantify and measure the atmosphere with continuous real-time data streams, which provide users and researchers access to pertinent air quality data through a cloud computing architecture. These data can be used by both members of the public and researchers. For example, AMS could be used to alert an elderly user with dementia about a gas leakage.

Further, AMS can find the effect of other atmospheric streams such as temperature and humidity on the user’s observed health and behavioral outcomes. This system monitors and analyses VOCs, which could help ensure that pregnant women breathe safe air, safeguarding not only their own health, but also that of their fetus(es). Particulate matter detection and warnings can help users to act on remediating the environment to avoid pulmonary complications in all age groups. Overall, the findings indicate that the multidimensional model of AMS is a step closer in considering a variety of pollutants and atmospheric characteristics that guide users toward a healthy lifestyle.

While the system can predict respiratory triggers, such as asthma attacks in vulnerable people, it is yet to be tethered to other health conditions (eg, proneness of a patient with diabetes to blood glucose variations due to air quality). AMS could certainly provide insight into such correlations and dramatically improve personal health.

## Appendix

Multimedia Appendix 1 Comparison of features and performance of COTS (Commercial-Off-The-Shelf) AQI sensor systems to AMS.

Multimedia Appendix 2 Summary of recent AQI studies as compared to the study presented in this work.

## Abbreviations

AMS: Atmosome Measurement System
ANN: artificial neural network
API: application programming interface
AQI: Air Quality Index
AWS: Amazon Web Services
COTS: commercial-off-the-shelf
DASE: data analysis and storage engine
DCS: data collection system
EPA: Environmental Protection Agency
IoT: internet of things
PCB: printed circuit board
PID: photoionization detector
PM_2.5_: particulate matter with diameter of 2.5 μm or less
PM10: particulate matter with diameter of 10 μm or less
ppb: parts per billion
ppm: parts per million
REST: representational state transfer
SoC: system-on-chip
tVOC: total volatile organic compound
UI: user interface
