# Supplementary material for: Understanding “Atmosome”, the Personal Atmospheric Exposome: Comprehensive Approach
Source: JMIR Biomed Eng. 2021 Nov 23;6(4):e28920. doi: 10.2196/28920 (PMC11041466; doi:10.2196/28920)
Supplement: Multimedia Appendix 1 [file biomedeng_v6i4e28920_app1.pdf]

Feature\Product Line	AMS	Aeroqual [35]	TSI [36]	Xiaomi Qingping air detector [32]	Temtop M2000 [37]	BRWISSEN A18 Air Quality Monitor [38]	EG Air Quality Pollution Monitor [39]	BREATHE| Smart 2.Air Quality Tester [40]	uHoo Indoor Air Quality Sensor [41]
Number of indoor air quality streams	18	16	5	3	4	9	5	1	6
Number of indoor atmospheric streams	4	0	3	2	2	0	2	0	3
Sensor Technology/Technologies	MQ	GSS/NDIR/GSE/LPC/PID	OPC/NDIR/PID	Unknown	LPC/NDIR/ElectroChemical	NDIR/ElectroChemical	LPC/ElectroChemical	LPC	Unknown
Cost (C) / Price (P)	$107 (C)	$650 (P)	$621 (P)	$150 (P)	$216 (P)	$209 (P)	$95 (P)	$129 (P)	$329 (P)
Analysis Time	Instantaneous	varies due to air mass transport factors and concentration gradients	Some streams measured real time	Fast-responding	Unknown	Faster	Unknown	Instantaneous	Unknown
Simultaneous recording of readings	✓		✓	✓	✓	✓	✓		✓
REST APIs to access data	✓								
Portable	✓	✓	✓	✓	✓	✓	✓	✓	✓
Web based Interface	✓								
Trends analysis	✓			✓	✓	✓			
Runs on Cloud Native Application	✓								
Recommends remediation	✓							✓	✓
Links to other devices				✓					✓
Threshold breach alerts/alarms	✓		✓		✓	✓		✓	
Precalibration				✓	✓			✓	✓
Manual calibration	✓					✓	✓		
AQI dial/display (on Web/device/phone)	✓	✓	✓	✓		✓	✓	✓	
Pollutant dashboard	✓				✓	✓	✓		✓
Pollutants & Gases									
$PM_{2.5}$ Monitoring	✓	✓	✓	✓	✓	✓	✓	✓	✓
PM_{10} Monitoring	✓	✓	✓		✓	✓	✓		
CO_2	✓	✓	✓	✓	✓	✓			✓
NO_2	✓	✓	✓						✓
O_3	✓	✓	✓						✓

CO	✓	✓	✓						✓
VOCs	✓	✓		✓		✓	✓		✓
LPG	✓								
NG	✓								
eCO_2	✓								
Ammonia	✓	✓	✓						
HCHO	✓	✓	✓		✓	✓	✓		
Hydrogen Sulfide	✓	✓	✓						
Methane	✓	✓							
Hydrogen	✓	✓							
Flammable gases	✓								
Alcohol	✓								
Chlorine		✓	✓						
Sulfur Dioxide		✓							
Perchloroethylene		✓							
NMHC (Non-Methane Hydrocarbon)		✓							
Aromatic compounds	✓								
PM (<= 1.0)						✓			
PM (>= 10)							✓		
Atmospheric streams									
Pressure	✓		✓						✓
Temperature	✓		✓	✓	✓		✓		✓
Altitude	✓								
Humidity	✓		✓	✓	✓		✓		✓

Table 1. Comparison of features and performance of COTS (Commercial-Off-The-Shelf) AQI sensor systems to AMS
