# Supplementary material for: Understanding “Atmosome”, the Personal Atmospheric Exposome: Comprehensive Approach
Source: JMIR Biomed Eng. 2021 Nov 23;6(4):e28920. doi: 10.2196/28920 (PMC11041466; doi:10.2196/28920)
Supplement: Multimedia Appendix 2 [file biomedeng_v6i4e28920_app2.pdf]

Study	Atmosome: A Comprehensive Approach to Understanding the Personal Atmospheric Exposome	Indoor air quality in two French hospitals: Measurement of chemical and microbiological contaminants	Volatile Organic Compounds (VOCs) in Conventional and High-Performance School Buildings in the U.S.	Characterization of Indoor Air Quality on a College Campus: A Pilot Study	Personal Exposure to Mixtures of Volatile Organic Compounds: Modeling and Further Analysis of the RIOPA Data	Indoor Air Quality in Green-renovated vs. Non-Green Low- Income Homes of Children Living in a Temperate Region of US (Ohio)	Indoor air pollution and exposure assessment of the gulf cooperation council countries: A critical review
Reference	AMS - Current Study	[42]	[43]	[44]	[45]	[46]	[47]
Pollutant Streams Measured	$PM_{2.5}$, PM_{10}, CO_2, NO_2, CO, VOCs, O_3, LPG, NG, eCO_2, H_2, NH_3, H_2S, CH_4, alcohol, formaldehyde, aromatic compounds, ambient parameters	VOCs, $PM_{2.5}$, PM_{10}, ambien`t parameters, virus, bacteria, fungi	Various types of VOCs	$PM_{2.5}$, $PM_{4,}$ PM_{10}, $PM_{100,}$ $CO_{2,}$ NOx, formaldehyde	Various types of VOCs	$PM_{2.5}$, black carbon, sulfur, VOCs, formaldehyde	$PM_{2.5}$, PM_{10}, VOCs, $CO_{2,}$ $SO_{2,}$ $NO_{2,}$, heavy metals
Pollution Sources	Non-ventilated rooms, commute cars, airplanes, kitchens, closed areas with cleaning services	Products, health care activities, and building activities used indoors in hospitals	Classrooms in recently renovated conventional and high-performance elementary schools	LEED certified, retrofitted, and conventional building types on a college campus	Vehicles, building materials, cleaning products, adhesives, repellents, chlorination	Green (protected with sealants) and non-green homes in low-income housing complexes	Household materials, kitchen activities, crowds
Detection Techniques	Low-cost sensors connected via microcontroller, processed in cloud	Carbopack, PUF, quartz filter, DNPH cartridge, Ecomesure, cyclonic air sampler	Electrochemi-cal analyzer, automated thermal desorber, gas chromatogra-phy, mass spectrometer	GK2.05 (KTL) aluminum cyclones, SKC button samplers and sampling pumps, TSI Q-trak, passive badges	Gas Chroma-tography, Mass Spectro-metry and passive samplers	HOBO logger, PTFE, membrane filters, PFC(perfluoro carbon) technique for airflow rate	Detection described by secondary GC-MS
Data Sampling Location	4 different domestic locations in 2 different countries were sampled	Two French hospitals were sampled in seven healthcare departments in different seasons	144 classrooms in 37 conventional and high-performance elementary schools were sampled	Three building types were sampled for a year	300 households in 3 cities in the US were sampled	Childrens' sleeping rooms in 800 apartments in a low-income multi-family housing complex were sampled	Web of Science, PubMed, Google search, WHO database were used to conduct the meta-analysis
Data Sampling Method	Cluster sampling	Cluster sampling	Cluster sampling	Stratified sampling	Convenience sampling	Convenience sampling	Convenience sampling

Data Sampling Duration	Continuous year-long data collection in US and India; ongoing collection in US	1 summer and 1 winter campaign, twice over 4 consecutive days	22 weeks of data collection efforts	6 sampling campaigns, each 48 hours, across 3 seasons	Sampled 2 times, 3 months apart	Measured over a five-day period, recording every five minutes	Data gathered from multiple papers
Statistical Analysis	Numerical and Time Series analysis & scatter plot and histogram analysis	Non-parametric tests with Bonferroni correction	One-way ANOVA and Kruskal Wallis (K-W) tests	Geometric means, standard deviation, log transformation, Fisher's Least Significant Difference	Extreme value distributions and mixture models	Linear mixed-effects models	Correlation analysis
Public Health Implications	Provides access to individual air quality data to analyze personal, geographical and demographical patterns	Air filtration in hospitals keeps them less polluted than regular indoor spaces	Additional research is needed to link IEQ and energy to health and performance in "green" buildings	Classrooms had more $PM_{2.5}$ than common areas, and green buildings had more pollution than conventional buildings	Exposure to VOCs is a large contributor to health issues across the body and diseases	It is important to further evaluate new, green and eco-friendly housing	Applying highly efficient particulate air filters is an urgent solution to reduce the emission of indoor PMs

Table 1. Summary of recent AQI studies as compared to the study presented in this work
